# Mlh1-Pms1 ATPase activity is regulated distinctly by self-generated nicks and strand discrimination signals in mismatch repair

**DOI:** 10.1093/nar/gkae1253

**Published:** 2024-12-19

**Authors:** Jonathan M Piscitelli, Scott J Witte, Yasmine S Sakinejad, Carol M Manhart

**Affiliations:** Department of Chemistry, Temple University, 1901 N. 13th St. Philadelphia, PA 19122, USA; Department of Chemistry, Temple University, 1901 N. 13th St. Philadelphia, PA 19122, USA; Department of Chemistry, Temple University, 1901 N. 13th St. Philadelphia, PA 19122, USA; Department of Chemistry, Temple University, 1901 N. 13th St. Philadelphia, PA 19122, USA

## Abstract

In eukaryotic post-replicative mismatch repair, MutS homolog complexes detect mismatches and in the major eukaryotic pathway, recruit Mlh1-Pms1/MLH1-PMS2 (yeast/human) complexes, which nick the newly replicated DNA strand upon activation by the replication processivity clamp, PCNA. This incision enables mismatch removal and DNA repair. Beyond its endonuclease role, Mlh1-Pms1/MLH1-PMS2 also has ATPase activity, which genetic studies suggest is essential for mismatch repair, although its precise regulatory role on DNA remains unclear. Here, we use an ATP-binding and hydrolysis-deficient yeast Mlh1-Pms1 variant to show that ATP hydrolysis promotes disengagement from Mlh1-Pms1-generated nicks, with hydrolysis in the Mlh1 subunit driving this activity. Our data suggest that the ATPase-deficient variant becomes trapped on its own endonuclease product, suggesting a mechanistic explanation for observations in genetic experiments. Additionally, we observed that Mlh1-Pms1 selectively protects DNA from exonuclease degradation at pre-existing nicks, which may act as strand discrimination signals in mismatch repair. Together, our findings suggest that Mlh1-Pms1 exhibits distinct behaviors on its own endonuclease products versus substrates with pre-existing nicks, supporting two distinct modes of action during DNA mismatch repair.

## Graphical Abstract

**Graphical Abstract:**
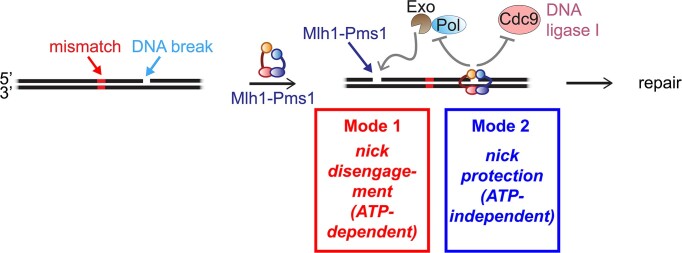

## Introduction

DNA mismatch repair is a highly conserved process that improves the overall fidelity of DNA replication by removing bases misincorporated by DNA polymerases in the nascent DNA strand (1). Consistent with this, defects in mismatch repair proteins are associated with increased mutation rates and, in humans, increased incidence of cancer (1,2).

In eukaryotes, the major mismatch repair pathway involves recognition of mismatches by either the Msh2-Msh6 or Msh2-Msh3 complex. These proteins then recruit the heterodimeric endonuclease Mlh1-Pms1 (in the yeast nomenclature, MLH1-PMS2 in mouse and human) which nicks the nascent DNA strand upon activation through interactions with the proliferating cell nuclear antigen (PCNA) protein. Mlh1-Pms1’s nick is critical for mismatch repair and several overlapping pathways have been suggested for how this nick is used to remove the mismatch (1,3–6). For exonuclease-dependent mismatch repair, the 5′-terminus of the Mlh1-Pms1 nick is used by Exo1 to begin excision and remove a segment of DNA that includes the mismatch. The resultant gap is then repaired by RPA, Polδ or Polϵ and a ligase. For exonuclease-independent repair, the 3′-end of the Mlh1-Pms1-generated nick is used as an initiation point for Polδ to synthesize DNA toward the mismatch, displacing a segment of the DNA strand that contains the mismatch as a flap, which is cleaved by the Rad27 flap endonuclease (5,7). Another pathway has been suggested where Mlh1-Pms1 can iteratively nick the nascent strand near the mismatch. In this scenario, the mismatch is removed either through diffusion of the oligonucleotides formed by the Mlh1-Pms1 repetitive nicking, by Exo1 exonuclease activity or strand displacement synthesis by Polδ (5,6,8,9).

These pathways all rely on Mlh1-Pms1 to generate a nick in the newly replicated DNA strand and not in the template strand. The mechanistic underpinnings for how this bias is achieved are unclear and have been highly sought-after largely through biochemical assays. Partially and fully reconstituted mismatch repair assays require plasmid-based substrates to both mimic chromosomal DNA where most of the genome is not in the vicinity of a DNA end, and to account for observations that MLH endonucleases have high affinity for and require large DNA substrates to be endonuclease active (6,10–17). In partially reconstituted mismatch repair assays dependent on the Mlh1-Pms1/PMS2 endonuclease activity, this activity is shown to have strand bias only when there is a pre-existing nick in the model plasmid substrate (14,15). In minimal systems using *Saccharomyces cerevisiae* and human proteins, it was shown that Mlh1-Pms1 requires PCNA to be loaded on DNA and that on DNA with a pre-existing discontinuity, nicking primarily occurs on the pre-nicked DNA strand (14,15,17).

Despite PCNA’s role in activating and directing repair *in vitro*, microscopy experiments in *S. cerevisiae* show that Mlh1-Pms1 foci do not co-localize with replication centers *in vivo* (18). It is unclear whether PCNA used in mismatch repair is a remnant of the replication fork or is loaded onto DNA by Replication Factor C (RFC) during repair. If the PCNA used in mismatch repair is loaded by RFC during repair, strand discontinuities on a newly replicated strand could act as PCNA loading sites. Regardless of what mechanistically pre-existing nicks are used for in DNA mismatch repair, both the leading and lagging strands can contain discontinuities (1,19,20) and pre-existing nicks have been established *in vivo* as being important for conveying strand bias. This was shown in experiments where the Cdc9 ligase was overexpressed and was antagonistic to mismatch repair in *S. cerevisiae* (21). These data suggest that prematurely removing discontinuities from newly replicated DNA eliminates the temporal window between DNA replication and mismatch repair, increasing mutation rates. It therefore follows that these discontinuities need to be preserved for the duration of time between DNA replication until post-replicative mismatch repair occurs to ensure the fidelity of the pathway. How DNA discontinuities are protected to promote strand discrimination and efficient mismatch repair has not been explored, but our data using purified Mlh1-Pms1 from *S. cerevisiae* suggest that Mlh1-Pms1 plays a direct role in maintaining DNA discontinuities as strand discrimination signals.

The Mlh1-Pms1 heterodimer has several notable biochemical properties that it uses to facilitate DNA mismatch repair. The homologous Mlh1 and Pms1 subunits contain conserved globular amino-terminal domains and globular carboxy-terminal domains that are connected via poorly conserved intrinsically disordered linker regions (22). The Mlh1 and Pms1 subunits primarily dimerize through their carboxy-terminal domains and this dimerization interface overlaps with the endonuclease site, which is primarily located in the carboxy-terminal domain of the Pms1 subunit (23). Mlh1-Pms1’s endonuclease activity has been shown previously to be activated mainly via interactions between the carboxy-terminal domain of Pms1 and PCNA, and has recently been shown to require a conserved motif in Mlh1’s disordered linker region that may interact with DNA, promote a necessary conformational change in Mlh1-Pms1 or interact with another mismatch repair factor (16,24). The globular amino-terminal domains of each subunit contain conserved ATPase sites (25–27). Although, ATP has been shown to not be absolutely required for endonuclease activity *in vitro*, it has been shown to have a stimulatory effect on this activity (13,28) and to promote transient dimerization between the Mlh1 and Pms1 subunits via the amino-terminal domains (25,26,29).

Previous research on the ATPase activities of *S. cerevisiae* Mlh1-Pms1 suggests that ATP binding is critical for mismatch repair *in vivo*, but the requirements for ATP hydrolysis are complex (25,27). In a study examining the role of ATP binding in yeast Mlh1-Pms1 activities, a conserved asparagine in the ATPase motif of each subunit was mutated to alanine: asparagine N35 in Mlh1 and asparagine N34 in Pms1 (using conventional codon numbering). This asparagine is essential for coordinating a divalent metal ion, which is necessary for ATP binding, as shown in *Escherichia coli* (27,29). In yeast, when this asparagine-to-alanine mutation was introduced in the Mlh1 subunit (*mlh1N35A*), the mutation rate increased by ∼9800-fold in a Lys^+^ reversion assay using a haploid strain (27). Introducing the same mutation in the Pms1 subunit (*pms1N34A*) led to a ∼11 000-fold increase in mutation rate in the same assay. For comparison, an *mlh1Δ* strain shows a similar ∼11 000-fold increase in mutation rate, while a *pms1Δ* strain results in a ∼9400-fold increase. In a study investigating the role of hydrolysis, a conserved glutamic acid in the ATPase motifs of Mlh1 and Pms1, which affects hydrolysis but not ATP binding, was mutated to alanine, both independently and together (25). When Mlh1’s ability to hydrolyze ATP was impaired (*mlh1E31A*), the mutation rate increased ∼300-fold relative to a wild-type strain in a *hom3-10* reversion assay and ∼7-fold in a canavanine resistance assay. When Pms1 was able to bind ATP but not hydrolyze it (noted as *pms1E61A* by the authors, though *pms1E30A* using the conventional start codon), the mutation rate increased ∼19-fold compared with wild type in the *hom3-10* assay and was unaffected in the canavanine resistance assay. In the double mutant (*mlh1E31A pms1E61A*), the mutation rates increased to ∼700-fold above wild type in the *hom3-10* reversion assay and ∼26-fold above wild type in the canavanine resistance assay. For reference, *mlh1Δ* and *pms1Δ* strains each increased mutation rates ∼1200- and ∼30-fold in the *hom3-10* reversion and canavanine resistance assays, respectively. In both the yeast system and in *E. coli*, ATP binding by MLH proteins has been shown to promote dimerization between the globular amino-terminal domains (25,26). These results suggest that ATP hydrolysis activity in both Mlh1 and Pms1 subunits is essential for Mlh1-Pms1’s function *in vivo*, with ATP binding in both subunits contributing to a synergistic effect.

Atomic force microscopy experiments and partial proteolysis assays using purified Mlh1-Pms1 have revealed significant conformational changes induced by ATP binding and hydrolysis via the intrinsically disordered regions (27,30). *In vitro* biochemical assays using yeast and *E. coli* homologs suggest that ATP hydrolysis regulates the interactions between the protein and DNA (28). These findings are consistent with assays involving yeast, human and bacterial homologs, which have revealed that Mlh1-Pms1’s ATPase activity is stimulated by DNA (16,28,29), and further enhanced by interactions with PCNA (16,28). Although not directly observed, the proposed large conformational changes in response to ATP binding and hydrolysis are believed to facilitate a recycling mechanism wherein Mlh1-Pms1 cleaves DNA in mismatch repair, dissociates or disengages from the nick during ATP hydrolysis and subsequently undergoes reactivation to repeatedly nick DNA. However, the mechanisms by which these signals are transmitted within the protein and how ATPase deficiencies lead to failures in this process remain unclear as iterative nicking does not seem to be required for efficient mismatch repair (6,8,9,28). Due to this, the role of Mlh1-Pms1’s ATPase activity and why it is critical for mismatch repair is unclear.

In this study, we use a purified yeast Mlh1-Pms1 variant deficient in ATP binding and hydrolysis to demonstrate that ATP hydrolysis promotes the release of Mlh1-Pms1 from its own endonuclease product. Experiments with point mutations in either the Mlh1 or Pms1 subunit revealed that this activity is primarily driven by ATP hydrolysis in the Mlh1 subunit. Exonuclease protection assays further showed that the Mlh1-Pms1 mutant deficient in ATPase activity in both subunits becomes trapped at its endonuclease sites. Additionally, we found that both the wild-type and ATPase-deficient Mlh1-Pms1 shield DNA from exonuclease degradation at pre-existing nicks that may function as strand discrimination markers during mismatch repair. Overall, our findings indicate that Mlh1-Pms1 responds differently to its own nicks compared with pre-existing nicks, suggesting two distinct modes of action in the DNA mismatch repair pathway. Furthermore, our investigation suggests a plausible explanation for the observed *in vivo* defects in ATPase mutants—although these mutants retain the ability to nick DNA, they become trapped at the nick site and are unable to dissociate. Consequently, this impairs access to the nick by downstream factors required for mismatch removal.

## Materials and methods

### Proteins and DNA substrates used in this study

Wild-type yeast Mlh1-Pms1 was expressed and purified as described previously (31). Mlh1-Pms1 ATPase mutations (*mlh1N35A-pms1N34A, mlh1N35A-Pms1* and *Mlh1-pms1N34A*) were generated by Q5 mutagenesis (NEB) using primers CMO175 and 176 for the mutation in Mlh1 using plasmid pMH1 and primers CMO177 and 178 for the mutation in Pms1 using plasmid pMH8 (Supplementary Table S1). Plasmids pMH1 and pMH8 that express yeast Mlh1 and Pms1, respectively, were a gift from Thomas Kunkel’s lab. Yeast PCNA and RFC were expressed and purified according to previous reports (32,33).

Unless otherwise noted 2.7 kb closed circular substrate is pUC18 and the 4.3 kb closed circular substrate is pBR322 (Invitrogen). Linear plasmid-based substrates were generated by digestion with HindIII restriction enzyme (NEB) according to the manufacturer’s instructions followed by heat inactivation. Substrates were then isolated by *P*-30 spin-column (Bio-Rad). Plasmid-based substrates with nicks were generated by incubating with nicking restriction endonucleases purchased from NEB: Nt.BspQI (one nick), Nb.BsrDI (two nicks), Nb.BtsI (three nicks), Nt.BstNBI (four nicks) and Nb.BsrDI was combined with Nb.BtsI (five nicks) (Supplementary Figure S1) according to the manufacturer’s instructions followed by spin-column purification. Relaxed circularized DNA without nicks was generated incubating five units of Topoisomerase I (NEB) with the DNA for 24 h and deactivated according to the manufacturer’s instructions. In experiments using the 5.8 kb DNA substrate (pRS423), linear plasmid was generated by digestion with SalI-HF (NEB) followed by heat inactivated according to the manufacturer’s instructions. The 5.8 kb substrate with four nicks was further digested using Nb.BtsI (NEB) according to the manufacturer’s instructions, followed by heat inactivation.

Biotinylated 4.3 kb plasmid DNA with and without pre-existing nicks used for ATPase assays where PCNA is pre-loaded was generated as previously described with minor modifications (11,34). Briefly, 4.3 kb closed circular pBR322 was nicked in a 60 μl reaction containing 50 mM potassium acetate, 20 mM Tris-acetate, 10 mM magnesium acetate, 100 μg/ml bovine serum albumin (BSA) and 60 units of the restriction enzyme Nt.BspQI (NEB) at 37°C overnight. This restriction enzyme generates one nick on the plasmid. The nicked strand was then excised using 20 units of T7 exonuclease (NEB) by incubating for 1 h at 37°C. Phosphorylated biotin primer (1.5 μM), /5Phos/CCATCGCG/iBiodT/CCGCCATCTCCA (IDT), was annealed to 150 nM of single-stranded circular pBR322 in a 60 μl reaction containing 50 mM Tris–HCl (pH 7.5), 10 mM MgCl_2_ and 10 mM dithiothreitol (DTT) by incubating the reaction at 85°C for 6 min and then allowing to cool overnight. Annealed primers were then extended in a 60 μl reaction containing 50 mM Tris–HCl, 10 mM MgCl_2_, 1 mM ATP, 10 mM DTT, 30 units of T4 DNA polymerase (NEB), 1000 units of T4 DNA ligase (NEB) and 1 mM dNTPs at 37°C for 1 h. Enzymes were then deactivated at 70°C for 20 min.

### Endonuclease assays

Endonuclease reactions were performed as previously described (12,28,35). Briefly, 20 μl reactions were combined containing varying concentrations of either wild-type or mutant Mlh1-Pms1, with 3.8 nM DNA substrate, in a buffer containing 20 mM HEPES–KOH, 20 mM KCl, 1% glycerol, 0.2 mg/ml BSA, 2.5 mM MnSO_4_ and 0.50 mM ATP (final concentrations). Reactions were incubated at 37°C for 60 min and stopped using stop mix [final concentrations: 1% sodium dodecyl sulfate (SDS), 14 mM ethylenediaminetetraacetic acid (EDTA), 0.96 units proteinase K]. Reactions were run on a 1.2% (w/v) agarose gel in a buffer containing 40 mM Tris, 20 mM acetate and 1 mM EDTA (1× TAE) for 1 h at 100 V. For endonuclease assays resolved under denaturing conditions, reactions were denatured with 30 mM NaOH, 1 mM EDTA, 3% glycerol and 0.02% bromophenol blue (final concentrations), then heated to 70°C for 5 min, and placed on ice for 3 min. Denatured endonuclease products were then resolved on a 1% (w/v) agarose gel containing 30 mM NaCl and 2 mM EDTA, in a solution of 30 mM NaOH and 2 mM EDTA at 50 V for 2.5–3 h. Denaturing gels were neutralized with 0.5 M Tris–HCl (pH 7.5) for 30 min. All gels were stained, visualized and quantified as described for electrophoretic mobility shift assays.

### Exonuclease inhibition assay

For exonuclease protection assays where Mlh1-Pms1 generates a nick, 3.8 nM 2.7 kb supercoiled pUC18 was combined with varying concentrations of wild-type or mutant Mlh1-Pms1, along with 100 nM RFC, and 500 nM PCNA in a buffer containing 20 mM HEPES–KOH, 20 mM KCl, 1% glycerol, 0.2 mg/ml BSA, 2.5 mM MnSO_4_ and 0.25 mM ATP. The reaction was incubated at 37°C for 1 h to promote endonuclease activity. Following this incubation, two units of T7 exonuclease (NEB) were added. Reactions were incubated at 37°C for an additional 30 min and stopped using stop mix (final concentrations: 1% SDS, 14 mM EDTA, 0.96 units proteinase K) as described above. Reactions were then incubated at 70°C for 30 min to stop the exonuclease reaction. Reactions were then analyzed by 1% (w/v) agarose gel resolved in 1× TAE at 100 V for 60 min, stained, visualized and quantified as described above.

For exonuclease protection assays on substrates with a pre-existing nick, 3.8 nM of 2.7 kb plasmid DNA containing one nick (generated with Nt.BspQI) was incubated with 0, 50 or 100 nM Mlh1-Pms1 in a buffer containing 50 mM potassium acetate pH 7.9, 20 mM Tris-acetate, 10 mM magnesium acetate and 100 μg/ml BSA for 10 min at room temperature. Two units of T7 exonuclease was then added and reactions were incubated for 30 min at 37°C and stopped using 1% SDS, 14 mM EDTA and 0.96 units proteinase K (final concentrations) for 10 min. Reactions were then analyzed by 1% (w/v) agarose gel resolved in 1× TAE at 100 V for 60 min, stained, visualized and quantified as described above.

### Electrophoresis mobility shift assays

DNA substrates (3.8 nM final concentration) were combined in 20 μl reactions on ice with varying concentrations of wild-type or mutant Mlh1-Pms1 in buffer A, which contains 20 mM HEPES–KOH, 0.1 mM DTT, 2 mM MgCl_2_, 40 μg/ml BSA and 6% glycerol (final concentrations). No KCl or NaCl was added to the reaction, unless otherwise noted. Where indicated, 500 nM of PCNA or 0.5 mM ATP was also included. Reactions were incubated for 10 min at room temperature and loaded into a 1% (w/v) agarose gel in 1× TAE that was prechilled for 1 h at 4°C. The gel was resolved on ice for 2 h at 45 V and stained in 300 ml of 1× TAE containing a final concentration of 10 μg/ml ethidium bromide.

### ATPase assays

ATPase assays in solution were performed as previously described and analyzed by thin layer chromatography (TLC) (16,26,27). Briefly, 10 μl reactions were combined containing 400 nM Mlh1-Pms1, 3.8 nM 4.3 kb pBR322 DNA substrate, 500 nM PCNA where indicated and 967 pmol [γ-^32^P]-ATP (Perkin Elmer) in a buffer containing 20 mM HEPES–KOH pH 7.5, 2.0 mM MgCl_2_, 20 mM KCl, 1% glycerol and 40 μg/ml BSA (final concentrations). Reactions were incubated for 45 min at 37°C. After incubating, 1 μl of reaction was spotted onto a polyethylenimine cellulose plate (Sigma–Aldrich). Plates were developed for 1 h using a solution of 1 M formic acid and 0.8 M LiCl_2_ and visualized by phosphorimaging.

For ATPase experiments on immobilized DNA, 20 μl of a solution containing 76 fmol of biotinylated closed circular 4.3 kb pBR322 in a buffer containing 20 mM HEPES–KOH pH 7.5, 20 mM KCl, 1% glycerol, 1 mM DTT and 0.2 mg/ml BSA was combined with 30 μl of streptavidin agarose resin (Thermo Fisher Scientific). The reactions were incubated on an agitator at 23°C for 30 min to bind the biotinylated DNA to the streptavidin-conjugated beads. Samples were washed three times with 30 μl, each wash, of a wash buffer containing 2 mM HEPES–KOH pH 7.5, 2 mM KCl, 0.1% glycerol and 0.02 mg/ml BSA. After the final wash, the supernatant is removed and 20 μl of a solution containing 10 pmol of PCNA and 2 pmol of RFC in a buffer containing 20 mM HEPES–KOH pH 7.5, 2.0 mM MgCl_2_, 100 μM ATP, 20 mM KCl, 1% glycerol and 40 μg/ml BSA (final concentrations) was added to the immobilized DNA and incubated at 37°C for 1 h. Reactions were then washed three times with the wash buffer above. The immobilized PCNA-loaded DNA was then divided into two portions. Each portion was then resuspended with 20 μl of a solution containing 100 mM NaCl, 50 mM Tris–HCl, 10 mM MgCl_2_ and 100 μg/ml of BSA (final concentrations). To the portion that is nicked by Nt.BstNBI (NEB) to generate the four nicked substrate, 20 units of the restriction enzyme were added and reactions were incubated for 1 h at 37°C. The reactions were then washed three times as previously described. To the immobilized PCNA-bound DNA, 10 μl of a solution containing 4 pmol Mlh1-Pms1, 967 pmol [γ-^32^P]-ATP (Perkin Elmer) and a buffer containing 20 mM HEPES–KOH pH 7.5, 2.0 mM MgCl_2_, 20 mM KCl, 1% glycerol and 40 μg/ml BSA (final concentrations) were added. Reactions were incubated for 45 min at 37°C, after which 1 μl of reaction was resolved and imaged by TLC as described above.

### Data quantification and statistical analyses

All gels and TLC plates were imaged using a Sapphire Biomolecular Imager (Azure) and quantified using ImageJ software. In endonuclease experiments assayed by native agarose gel, nicking activity is quantified as the amount of DNA converted to nicked plasmid from supercoiled substrate. The amount of nicked or relaxed plasmid in negative controls was subtracted as background. For endonuclease experiments assayed by denaturing agarose gel, because Mlh1-Pms1 endonuclease activity is nonspecific across substrate molecules, nicking activity is quantified as the amount of substrate DNA lost in reactions relative to negative controls. In electrophoretic mobility shift assays on plasmid DNA, because Mlh1-Pms1 binds to large DNA molecules as oligomeric complexes (10–12,18,36–38) and contorts their shape (12,38), the amount of DNA bound was quantified as the loss of DNA in the substrate band relative to negative controls.

All experiments were performed in at least duplicate, with the number of replicates given for each experiment. When data are presented as a plot, the mean is shown with error bars representing the standard deviation (SD) between experiments.

## Results

### Mlh1-Pms1 uses the ATPase activity in Mlh1 to nick DNA substrates more than once

Although *in vivo* experiments suggest that Mlh1-Pms1’s ATPase activity is essential for its mismatch repair functions (25,27), and ATP binding and hydrolysis are known to promote conformational changes in the protein (26,29,30), the precise mechanistic role of these activities in mismatch repair remains to be fully established. It has been suggested, though not directly demonstrated, that Mlh1-Pms1 can repetitively nick DNA, with this turnover hypothesized to result from the recycling of Mlh1-Pms1 on DNA—a process promoted by the protein’s ATPase activity (6,8,9,28). Support for iterative rounds of endonuclease activity driven by Mlh1-Pms1 ATPase activity includes evidence of Mlh1-Pms1 ATPase stimulation by DNA, the observed large conformational shifts in the protein in the presence of ATP and the apparent lowering of affinity for oligonucleotides in the presence of ATP in some assays (16,28–30,39,40). Additionally, in single molecule experiments, mutants lacking portions of the intrinsically disordered regions, which display ATPase defects, create fewer nicks on DNA substrates than wild-type Mlh1-Pms1 (28).

To directly test for Mlh1-Pms1’s ability to use its ATP hydrolysis function to nick the same DNA substrate more than once, we generated a mutant version of Mlh1-Pms1 that is defective in ATP binding and hydrolysis in both subunits, mlh1N35A-pms1N34A (25,27). In *in vivo* experiments measuring mismatch repair efficiency in bakers’ yeast, both a *mlh1N35A* and a *pms1N34A* strain displayed mutation rates similar to *mlh1Δ* and *pms1Δ* strains (27). In biochemical characterization assays for these mutants, the amino-terminal ATPase domains of either Mlh1 or Pms1 were isolated as truncations of either wild-type, mlh1N35A or pms1N34A mutants. In these experiments, both the isolated amino-terminal domain of Mlh1 with the N35A mutation and the isolated amino-terminal domain of Pms1 with the N34A mutation were deficient in both ATP binding and hydrolysis when measured directly (27).

**Figure 1.**
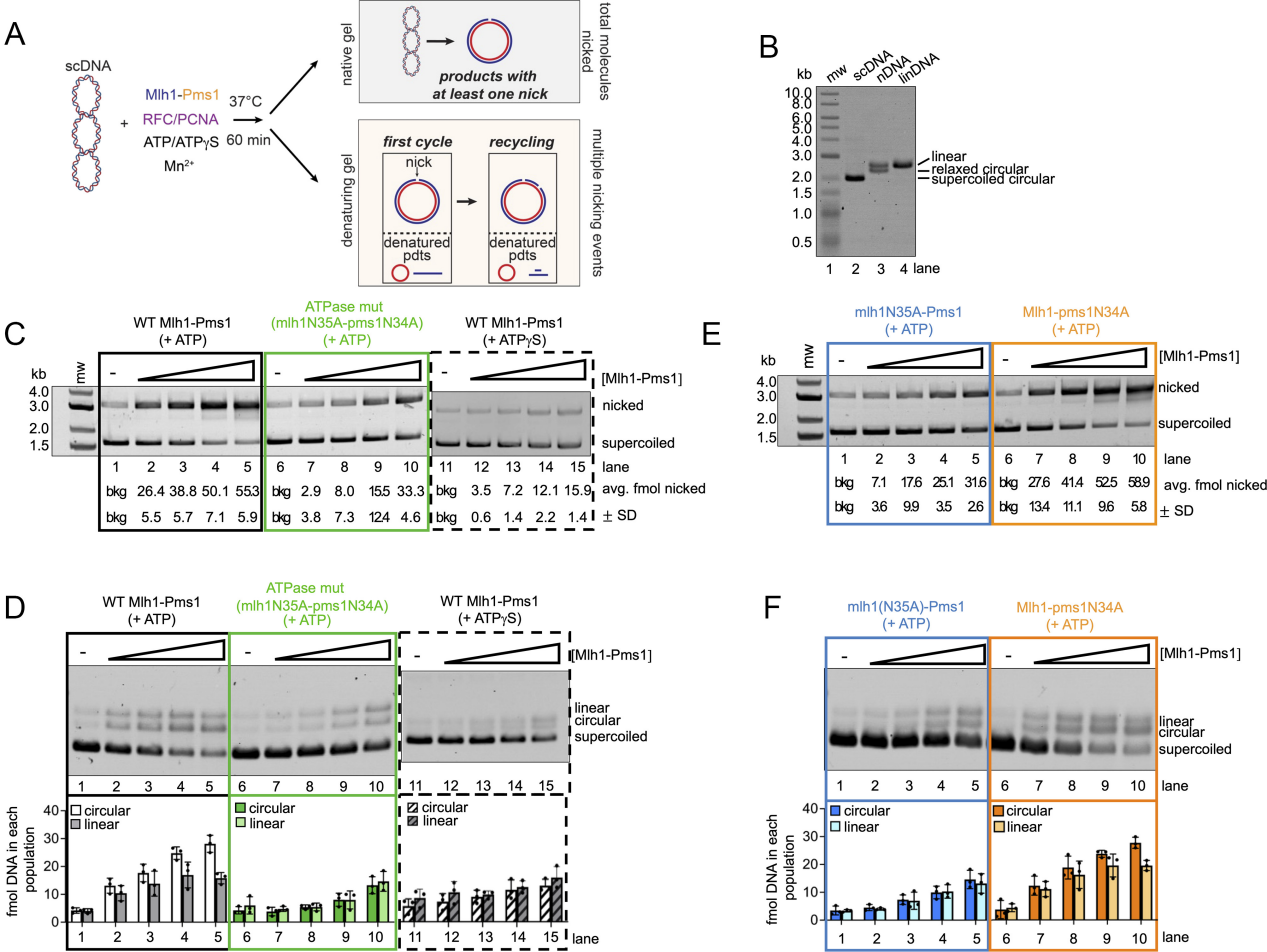
The ATPase activity in the Mlh1 subunit drives Mlh1-Pms1 endonuclease recycling on DNA. (**A**) Schematic for assay. Wild-type Mlh1-Pms1 or the indicated variant was incubated with 2.7 kb supercoiled pUC18 DNA (scDNA) along with 100 nM RFC, 500 nM PCNA and 0.5 mM ATP or 0.5 mM ATPγS as indicated in a buffer containing 20 mM KCl and 2.5 mM MnSO_4_ to probe for endonuclease activity in the absence of a mismatch and an MSH complex. See ‘Materials and methods’ section for additional details. Reaction products were split and analyzed by native agarose gel to measure the total number of molecules that are nicked at least once and denaturing agarose gel to measure whether substrates are nicked more than once and whether multiple nicking events are dependent on one another. (**B**) Control denaturing gel demonstrating migration of possible DNA products produced in this assay. Lane 2 contains 2.7 kb scDNA (pUC18), lane 3 contains 2.7 kb DNA that contains one nick (Nt.BspQI) and lane 4 contains 2.7 kb DNA linearized with HindIII. (**C**) Endonuclease assay measuring population of 2.7 kb scDNA (135 ng) substrates that are nicked at least once by wild-type Mlh1-Pms1, mlh1(N35A)-pms1(N34A) ATPase mutant, or the wild-type protein in the presence of 0.5 mM ATPγS. Either 0, 25, 50, 100 and 200 nM of wild-type Mlh1-Pms1 or ATPase mutant was incubated with the scDNA, along with 500 nM PCNA and 100 nM RFC. The amount of DNA nicked was quantified by calculating the proportion of the DNA in the nicked product band relative to the total amount of DNA in each lane (*n* = 3). (**D**) Denaturing analysis of material in panel (C). The density of the linear and circular band in each lane was measured and calculated as a proportion in fmol relative to the density in the negative control lanes 1, 6 and 11 which represent the total fmol of DNA in each experimental lane (*n* = 3). (**E**) The number of supercoiled plasmids nicked by wither mlh1N35A-Pms1 or Mlh1-pms1N34A (*n* = 3) was measured identically to how performed in panel (C). **(F)** Denaturing analysis of material in panel (E). Quantifications were performed as described in panel (D) (*n* = 3).

To determine whether the mlh1N35A-pms1N34A ATPase mutant is capable of recycling and promoting multiple nicking events, we performed an endonuclease assay that measures both the total number of DNA molecules nicked by the endonuclease and whether more than one event likely occurs per substrate on 2.7 kb plasmid DNA (Figure 1A). It should be noted that, to observe Mlh1-Pms1 endonuclease activity in the absence of a mismatch and/or an MutS homolog (MSH) complex, we and others typically perform assays at low ionic strength (∼20 mM KCl) to isolate this activity (11–13,15,24,28). Using near-physiological salt conditions, we observe virtually no endonuclease activity on substrates lacking a mismatch and an MSH complex (Supplementary Figure S1A–C). Additionally, Mlh1-Pms1, like other mismatch repair factors, has been shown to bind to DNA ends (41–44), making circular plasmid substrates without DNA ends the preferred substrate for these experiments.

In this experiment, the total number of DNA molecules nicked are assessed as conversion of scDNA to nicked circular product (Figure 1A, top panel). In this analysis, we observed that the mlh1N35A-pms1N34A ATPase mutant exhibited a mild defect in endonuclease activity relative to the wild-type protein (Figure 1C, compare lanes 2–5 with 7–10).

To determine if the *mlh1N35A-pms1N34A* ATPase mutant is defective in nicking the same DNA substrate multiple times, we analyzed the reaction products from the assay in Figure 1C using a denaturing gel system. The native gel analysis in Figure 1C is limited in that one nick per substrate is sufficient to convert the substrate to a nicked product, but it does not indicate whether a DNA molecule is nicked more than once. In a denaturing gel, if scDNA is nicked once, the reaction products will migrate as a single-stranded closed circular DNA and a linear DNA molecule (Figure 1A and B). The band densities of the single-stranded circular form and the linear form will be equivalent if there is one nick per substrate and each density in femtomoles will be equivalent to the total femtomoles of scDNA in the reaction initially. If Mlh1-Pms1 nicks the same plasmid more than once, the band density of either the single-stranded closed circular form or the linear form will deplete because nicking is nonspecific on these substrates. We would expect that the linear form would at least partially preferentially deplete over the single-stranded closed circular form, because the reaction contains excess RFC, PCNA and ATP, so RFC is likely to use an initial Mlh1-Pms1-generated nick as a loading site. It has been shown previously that the combination of RFC and PCNA can partially bias Mlh1-Pms1 endonuclease activity to the nicked DNA strand (15,17). Consistent with this, we observe that when we analyze endonuclease reaction products generated by wild-type Mlh1-Pms1 in the denaturing system, we observe an apparent degradation of the linear DNA band (Figure 1D, lanes 1–5).

It should be noted that a single additional nick is sufficient to cause degradation of the linear DNA band in this assay, so while this approach can detect the occurrence of a second nick, it cannot explicitly confirm iterative nicking. Furthermore, if a second nick is close to the first on the same strand, the distance may be too short to affect the migration of the linear band, potentially obscuring detection of degradation. In contrast, a second nick further from the first will cause apparent degradation of the linear band. We also anticipate some background in this assay due to the initial random orientation of PCNA loading on scDNA. This could result in a plasmid molecule with PCNAs loaded in opposite orientations, potentially leading to nicks on either strand in the first nicking cycle. In such cases, subsequent nicking events may still be influenced by these initial nicks, but the observed increase in circular single-stranded product may not correlate directly with the decrease in scDNA substrate. Despite these limitations, this assay allows us to assess Mlh1-Pms1’s capacity for recycling on DNA.

When we performed the denaturing analysis with the mlh1N35A-pms1N34A ATPase mutant endonuclease products, the measured band density for the single-stranded circular form and linear form of DNA were nearly equivalent to one another in terms of the number of femtomoles of DNA; however, the number of femtomoles in each band was not equivalent to the amount of supercoiled starting material (Figure 1D, lanes 6–10). Because one band is not apparently depleting relative to the other, our data suggest that the mlh1N35A-pms1N34A mutant is not recycling. Because the amount of DNA migrating at the single-stranded circular position and the linear positions are not equivalent to the amount of supercoiled starting material, this suggests that although there may be multiple nicks on each substrate they are independent of one another and possibly caused by random orientations of PCNA during the first nicking cycle.

To determine if recycling was merely delayed in the mlh1N35A-pms1N34A mutant relative to the wild-type protein, we also performed the experiment and analyzed the reaction products after a 2-h incubation (Supplementary Figure S1D and E). This experiment also included an additional, higher concentration of Mlh1-Pms1 relative to the experiments in Figure 1. With the longer incubation, we still did not observe preferential depletion of one DNA strand relative to another when we analyze the mlh1N35A-pms1N34A ATPase mutant reaction products by denaturing analysis, suggesting that the mlh1N35A-pms1N34A ATPase mutant is defective in recycling after nicking DNA. In this analysis, the preferential depletion of one DNA strand relative to another for the wild-type protein was more subtle, which is likely due to increased random PCNA loading events independent of the nick sites due to the increased reaction time.

We also performed the experiment described in Figure 1A with wild-type Mlh1-Pms1 in the presence of ATPγS. It has been shown previously that clamp loading complexes load processivity clamps minimally in the presence of non-hydrolyzable analogs of ATP as ATP binding is required for clamp loading and hydrolysis is required for clamp closing and dissociation, suggesting that each RFC may be able to load one PCNA, but the clamp may be unstable on DNA in the presence of ATPγS (45). In these reactions, we observed minimal endonuclease activity overall (Figure 1C, lanes 11–5), consistent with minimal PCNA loading as PCNA is required to measure Mlh1-Pms1’s endonuclease activity on these substrates under the tested conditions (Supplementary Figure S1F). In the denaturing analysis, we observed no preferential depletion of one endonuclease product over another, suggesting that recycling is not occurring, similar to what we observed with the mlh1N35A-pms1N34A ATP hydrolysis mutant. However, it appears that a single cycle of nicking is taking place, as the femtomole quantities of the single-stranded circular form, the linear form and the total nicked product in the native gel analysis are equivalent (Figure 1D, lanes 11–15).

Previous biochemical research indicates that MutL homolog subunits exhibit asymmetric behavior when dimerized, whether forming a MutL homodimer with bacterial subunits or eukaryotic heterodimers (25,27,29). Specifically, for yeast Mlh1-Pms1, studies have shown that the truncated Mlh1 ATPase domain has a lower *K*_m_ compared with the truncated Pms1 ATPase domain. Additionally, in kinetic ATPase assays, the Pms1 ATPase domain exhibits a higher *k*_cat_ value than the Mlh1 ATPase domain (27).

We next aimed to identify which Mlh1-Pms1 subunit’s ATPase activity primarily drives recycling. To address this, we expressed and purified mutant variants: one with impaired ATPase activity solely in the Mlh1 subunit (mlh1N35A-Pms1), and another with deficiency solely in the Pms1 subunit (Mlh1-pms1N34A). We then measured endonuclease activity in a population-based assay to determine the total number of DNA molecules nicked by the Mlh1-Pms1 ATPase variants. Our findings revealed that the Mlh1-pms1N34A mutant displayed activity comparable to the wild type. In contrast, the mlh1N35A-Pms1 mutant exhibited slightly inhibited activity, with nicking efficiencies similar to those of the mlh1N35A-pms1N34A mutant (see Figure 1E, lanes 1–5 compared with Figure 1C, lanes 6–10). By analyzing the reaction products using the denaturing analysis, which allows us to measure additional nicking events, we similarly found that the mlh1N35A-Pms1 mutant was inhibited for promoting multiple rounds of endonuclease activity, whereas the Mlh1-pms1N34A mutant was proficient in this activity (Figure 1F). These results suggest that ATPase activity in the Mlh1 subunit is critical for supporting Mlh1-Pms1 recycling on DNA after nicking, whereas Pms1’s ATPase activity is less essential for this process.

### Mutants defective in ATPase activity are trapped after nicking DNA

The motifs facilitating ATPase activity in MutL homologs are highly conserved across organisms (26). Consistent with an important biological significance, missense mutations in the ATPase region of human MLH1 have been identified in patients with Lynch syndrome, a hereditary cancer syndrome associated with mismatch repair defects (46–48). Included in this set are mutations to residue N38 in human MLH1 that aligns with N35 in yeast Mlh1. Mutations adjacent to the ATPase motif also have been shown to affect the cellular localization of mammalian MLH1 and are associated with breast cancers (49). Because the ATPase site in Mlh1 is biologically significant across organisms (27), and our findings in Figure 1 indicate that an Mlh1-Pms1 ATPase mutant retains functional endonuclease activity, despite failures to recycle, we aimed to elucidate the consequences of impaired recycling to better explain *in vivo* defects.

**Figure 2.**
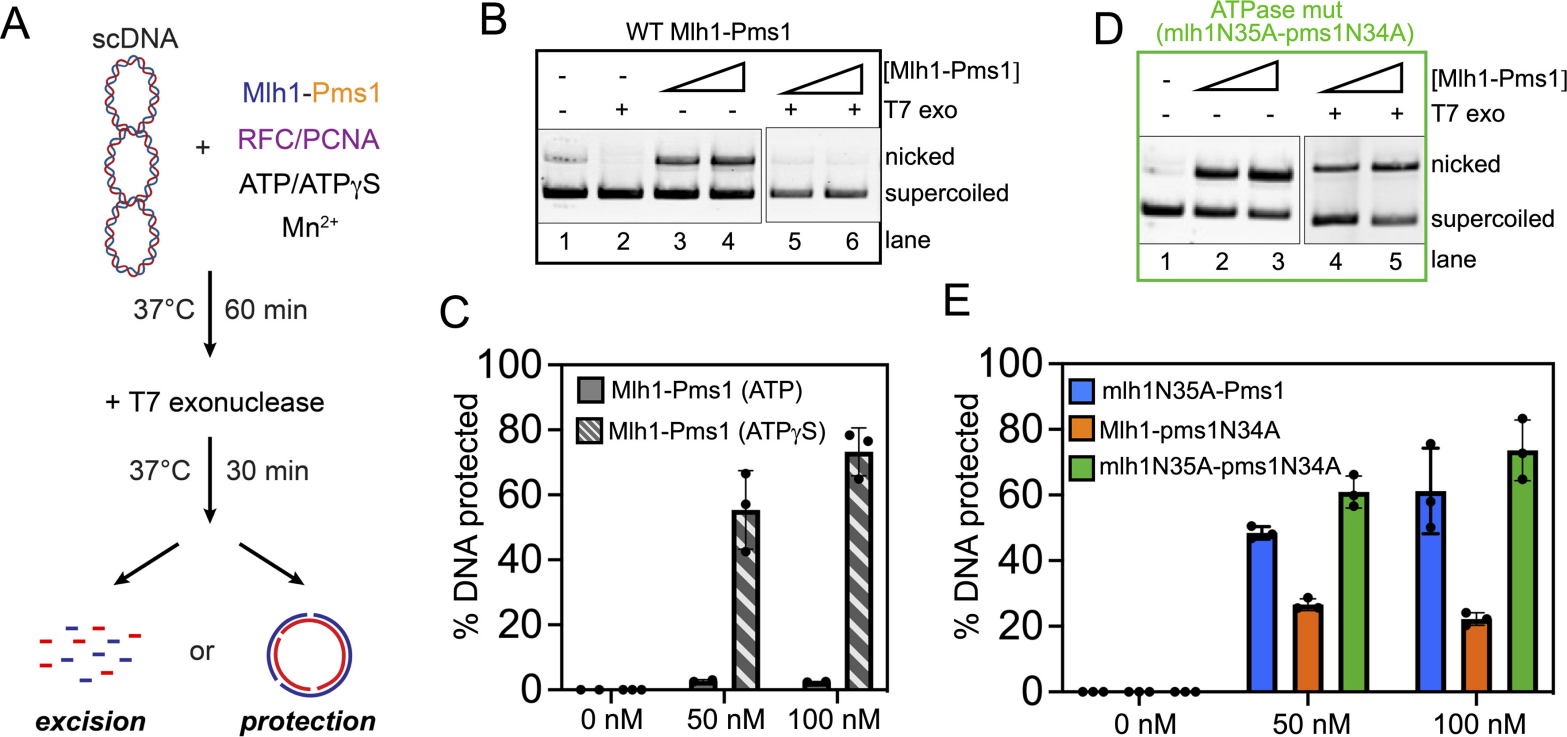
An Mlh1-Pms1 ATPase mutant is trapped at DNA nicks. (**A**) Schematic for assay. Either 0, 50 or 100 nM of wild-type Mlh1-Pms1 or mlh1N35A-pms1N34A ATPase mutant was incubated with 2.7 kb pUC18 scDNA, where indicated, in the presence of 500 nM of PCNA, 100 nM RFC and 0.50 mM ATP (or 0.50 mM ATPγS) under conditions that promote endonuclease activity in a buffer containing 20 mM KCl and 2.5 mM MnSO_4_. After incubation, T7 exonuclease was added, where indicated, which degrades the substrate in the absence of Mlh1-Pms1 (Supplementary Figure S2A and B). See ‘Materials and methods’ section for complete description. (**B**) T7 exonuclease protection products for wild-type Mlh1-Pms1 in the presence of ATP were analyzed by agarose gel. (**C**) The proportion of DNA protected from T7 exonuclease degradation was calculated by quantifying the amount of nicked DNA for each Mlh1-Pms1 concentration in the presence of T7 exonuclease relative to the amount without T7 exonuclease. For wild-type Mlh1-Pms1 in the presence of ATP, *n* = 2. For wild-type Mlh1-Pms1 in the presence of 0.5 mM ATPγS in place of ATP, *n* = 3. See Supplementary Figure S2C for sample raw data for the ATPγS condition. (**D**) Agarose gel analysis of reaction products for the mlh1N35A-pms1N34A ATPase mutant in the presence of ATP. (**E**) The proportion of DNA protected from T7 exonuclease degradation for the mlh1N35A-pms1N34A mutant and mlh1N35A-Pms1 and Mlh1-pms1N34A single mutants (see Supplementary Figure S2D and E for sample raw data for single mutants) was calculated as described for panel (C). For all variants, *n* = 3.

To determine the fate of the mlh1N35A-pms1N34A mutant on DNA, we conducted a nonspecific nicking reaction on scDNA, allowing either the wild-type or the ATPase mutant protein to nick the DNA (Figure 2A). Subsequently, T7 exonuclease was introduced to degrade DNA in the 5′ to 3′ direction, initiating at nicks generated by Mlh1-Pms1 (Supplementary Figure S2A and B). We found that when the wild-type protein was permitted to nick the DNA in the presence of ATP, T7 exonuclease nearly completely degraded the DNA molecules nicked by wild-type Mlh1-Pms1 (Figure 2B and C). This suggests that after nicking the DNA, Mlh1-Pms1 can disengage from the nicks. In contrast, reactions with the mlh1N35A-pms1N34A ATPase mutant showed inhibited degradation by the exonuclease (Figure 2D–E). To determine if the mutations themselves caused the change in behavior on endonuclease products, we also performed this assay with wild-type Mlh1-Pms1 in the presence of ATPγS (Figure 2C, and Supplementary Figure S2C). When the non-hydrolyzable ATP analog was included in the endonuclease reaction, although less DNA is nicked due to inefficiency of PCNA loading, we observed that the proportion of DNA protected from T7 exonuclease degradation was similar to experiments where the mlh1N35A-pms1N34A ATPase mutant was included. These results indicate that the mlh1N35A-pms1N34A ATPase mutant not only fails to recycle *in vitro* but also becomes trapped at DNA nicks, protecting the entire substrate from T7 degradation. Despite its competence in endonuclease activity, the protein remains tethered at the nick site, providing a possible explanation for the observed defects both in yeast genetic assays and the association with Lynch syndrome (25,27,47,48).

We also performed this experiment with the mlh1N35A-Pms1 and Mlh1-pms1N34A single mutants to determine if one subunit preferentially drives endonuclease product disengagement (Figure 2E, and Supplementary Figure S2D and E). Consistent with the data in Figure 1, we found that the mlh1N35A-Pms1 mutant protected nicked DNA products to a similar extent as the mlh1N35A-pms1N34A double mutant. The Mlh1-pms1N34A mutant protected nicked products from excision significantly more than the wild-type protein under these conditions, but less than the mlh1N35A-Pms1 mutant and less than the double mutant with both subunits defective in ATPase activity. Interestingly, the contributions from each subunit did not appear to be strictly additive. This suggests that while the Mlh1 subunit may play a greater role in disengagement from the nicked DNA product, ATP hydrolysis by both subunits and their combined effects are required for efficient function. This finding aligns with data from *E. coli* MutL, where ATP binding by the subunits promotes dimerization of the amino-terminal domains, and ATP hydrolysis appears to drive their dissociation, potentially leading to DNA release from a MutL clamp that opens upon ATP hydrolysis (26,29,30). Our data suggest that ATP hydrolysis in each subunit may contribute to this cycle, suggesting a coordinated mechanism of DNA binding and release.

**Figure 3.**
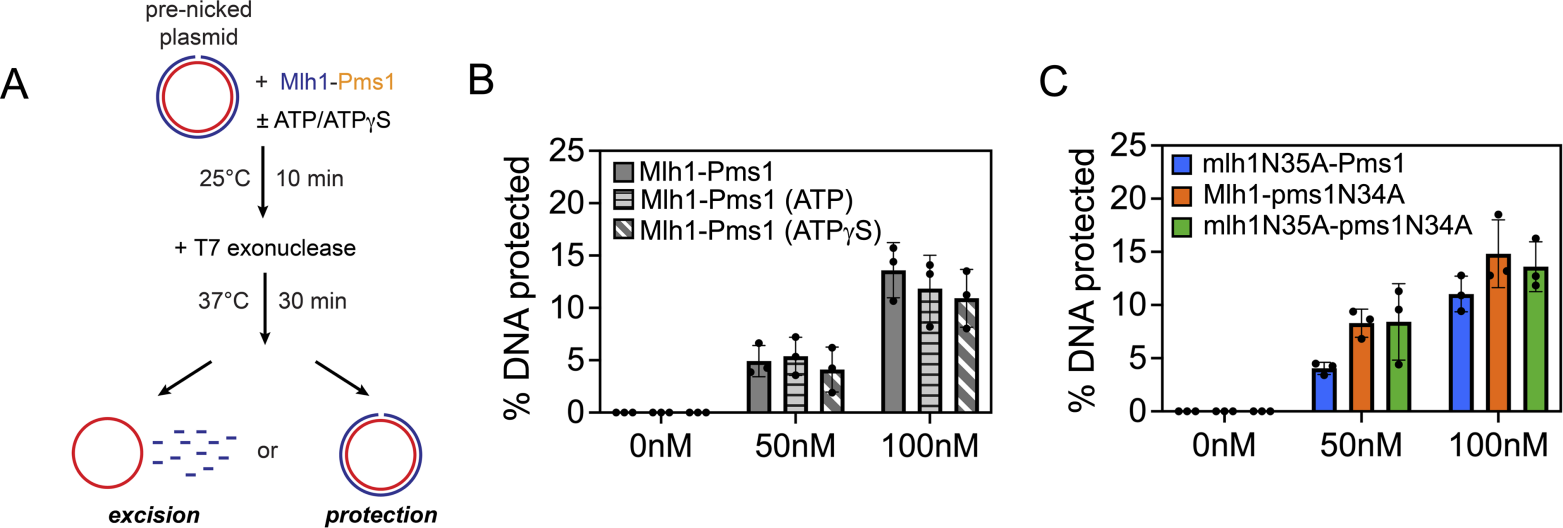
Mlh1-Pms1 exhibits distinct behavior on pre-existing nicks compared with endonuclease products. (**A**) A 2.7 kb pUC18 plasmid with a single pre-existing nick was combined with wild-type Mlh1-Pms1 or an ATPase variant in the presence or absence of 0.50 mM ATP or 0.50 mM ATPγS. No KCl or NaCl was added to the reaction to promote DNA binding and to not interfere with the T7 exonuclease. See ‘Materials and methods’ section for additional details. (**B**–**C**) The proportion of DNA protected from T7 exonuclease degradation was measured as in Figure 2 relative to negative controls where T7 degrades the pre-nicked DNA strand. Raw data are included in Supplementary Figure S2F and G. For all conditions, *n* = 3.

We aimed to discern whether the trapping of the mlh1N35A-pms1N34A ATPase mutant at a DNA nick resulted from a specific conformation post-DNA incision, or if pre-existing single-strand breaks in the DNA induced a similar effect. In other words, does Mlh1-Pms1 display different behaviors on nicks that are Mlh1-Pms1 endonuclease products relative to pre-existing nicks in the substrate. To test this, we generated a DNA substrate with a single pre-existing single-strand break and pre-bound either the wild-type or mlh1N35A-pms1N34A ATPase mutant protein to the DNA (Figure 3A). We omitted RFC and PCNA from the reaction to not induce endonuclease activity. We then added an amount of T7 exonuclease that in the absence of Mlh1-Pms1 degrades the entirety of the nicked strand (Supplementary Figure S2A and B). Using the DNA with the pre-existing nick, we observed that both the wild-type and the mlh1N35A-pms1N34A ATPase mutant protein protected the DNA from T7 exonuclease degradation to similar extents (Figure 3B and C). We also observed that ATP, ATPγS and assaying the variants where either Mlh1 or Pms1 is impaired for ATPase activity (mlh1N35A-Pms1 or Mlh1-pms1N34A) had very little effect on the proportion of DNA protected which is distinct from our observations in Figure 2 where the Mlh1-Pms1 complex generates this nick. It should be noted that pre-existing single-strand breaks in the DNA were introduced using commercially available restriction nicking endonucleases, which produce discontinuities containing a 3′-hydroxyl group and a 5′-phosphate group. Mlh1-Pms1 likely makes nicks with identical chemistries given that these nicks serve as substrates for polymerases and exonucleases in mismatch repair. Taking this inference into account along with the observation that the mlh1N35A-pms1N34A ATPase mutant and wild-type Mlh1-Pms1 protect pre-existing breaks to similar extents, the difference in protection observed in Figure 2 compared with Figure 3 is likely caused by Mlh1-Pms1 accessing a specific conformation after nicking the DNA and not simply through recognizing the DNA discontinuity. How Mlh1-Pms1 treats the nick it makes versus a pre-existing nick are therefore not equivalent and represent two modes of the protein (see ‘Discussion’ section). Together these data suggest that defects observed for mlh1N35A-pms1N34A ATPase mutants *in vivo* (27) may be caused by the protein making a nick to initiate mismatch removal, but failing to disengage from the nicking site preventing mismatch removal and allowing both the mismatch and the nick to persist.

### Mlh1-Pms1 shows minimal intrinsic preference for DNA with pre-existing nicks compared with unnicked DNA

Because wild-type Mlh1-Pms1 showed distinct behavior in protecting a pre-existing nick from T7 exonuclease degradation relative to its endonuclease product, we sought to determine if eukaryotic Mlh1-Pms1 specifically recognizes DNA discontinuities. Prior studies suggest that MLH endonucleases bind DNA cooperatively as oligomeric complexes (10–12,19,35–37). Consistent with MLH oligomer models, only large DNA substrates (∼1 kb) effectively support MLH endonuclease activity, and DNA affinity rises significantly with substrates of at least 500 bp (10–12). Additionally, as stated previously, MLH complexes have been shown to bind to free DNA ends (41–44), thus, despite plasmid substrates containing large tracts of DNA that can be bound nonspecifically, they are more suitable than oligonucleotides for Mlh1-Pms1 binding studies. Additionally, they allow us to compare to our experiments in Figures 1–3 which were performed on plasmid substrates.

**Figure 4.**
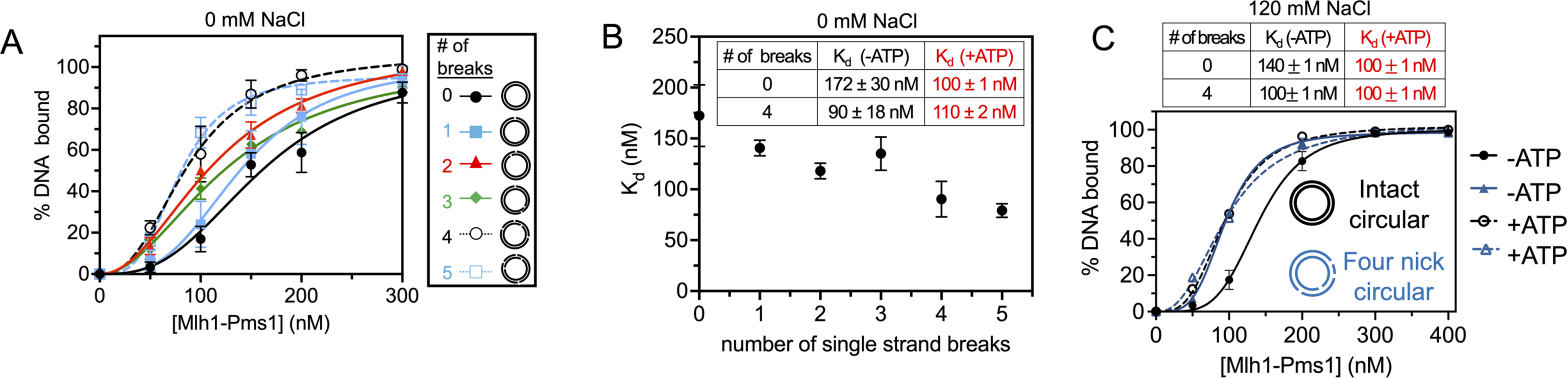
Mlh1-Pms1 has a minimal affinity preference of DNA with pre-existing nicks over unnicked DNA. (**A**) Mlh1-Pms1 DNA binding curves on 2.7 kb DNA substrates that are fully relaxed or containing a variable number of single-strand breaks with no additional NaCl added or ATP (see Supplementary Figure S3 for example of data and quantification and Supplementary Figure S4 for complete raw data). Single-strand breaks were generated according to the ‘Materials and methods’ section. Mlh1-Pms1 was titrated to final concentrations: 0, 50, 100, 150, 200 or 300 nM. For substrates with zero, two, three or five nicks, *n* = 3. For substrates with one or four nicks, *n* = 4. Data were fit to a sigmoidal function modeling cooperative binding. Hill coefficients were ∼3.0 for all curves. Representative primary data are in Supplementary Figure S4. (**B**) *K*_d_ values were obtained from the binding data in panel (A) and plotted relative to the number of nicks. (**C**) Mlh1-Pms1 binding curves on 2.7 kb circular DNA that are either fully relaxed or contain four nicks at 120 mM NaCl. Mlh1-Pms1 was titrated to final concentrations: 0, 50, 100, 200, 300 or 400. Dashed lines indicate 0.5 mM ATP was added into those reactions. For all substrates, *n* = 2. *K*_d_ table was generated from the graphed data in panel (C) using a sigmoidal function model.

To examine Mlh1-Pms1’s DNA binding specificity, we prepared 2.7 kb circular plasmids with varying numbers of nicks, ranging between zero and five. Our findings showed that although the approximate *K*_d_ values decreased slightly with increasing number of pre-existing nicks (Figure 4A and B, and Supplementary Figures S3 and S4), there was no more than a 2-fold difference in affinity between the fully intact and the five-nicked plasmids, suggesting minimal preference for nicked DNA. This assay was performed at 20 mM KCl to mimic conditions that support Mlh1-Pms1 endonuclease activity and T7 exonuclease protection used in Figures 1–3. When we increased the ionic strength to 120 mM NaCl in this assay, we did not observe a significant change in affinity or selectivity for a substrate with pre-existing nicks over continuous, intact DNA (Figure 4C).

Given that *E. coli* MutL can recognize DNA with 3′-resected ends in an ATP-dependent manner (44), we tested Mlh1-Pms1’s affinity for relaxed circular DNA and a four-nicked plasmid in the presence of ATP (Figure 4B and C). We did not observe significant changes in affinity on either substrate in the presence of ATP at low or near-physiological ionic strength. The estimated *K*_d_, value changed by no more than ∼1.7-fold under any condition and the raw electrophoresis mobility shift data (Supplementary Figure S4) do not suggest significant changes in affinity by direct observation. Together, these data suggesting that Mlh1-Pms1’s behavior on pre-existing nicks is not due to markedly higher affinity for these structures.

Since PCNA and DNA independently and synergistically enhance Mlh1-Pms1 ATPase activity (16,28,29), we also examined the effect of PCNA on Mlh1-Pms1’s affinity for single-strand breaks using a 2.7 kb linearized plasmid, which supports robust nonspecific *in vitro* endonuclease activity and allows PCNA to self-load without RFC. Due to PCNA’s sliding clamp nature, we measured only Mlh1-Pms1’s affinity for DNA and observed only minimal effects of PCNA and/or ATP on affinity for either substrate at low ionic strength (Supplementary Figure S5A–J and Q) and at near-physiological ionic strength (Supplementary Figure S5K–O and Q), suggesting that Mlh1-Pms1’s behavior on pre-existing nicks is not explicitly driven by selectivity for these structures.

Additionally, we tested the mlh1N35A-pms1N34A mutant’s ability to recognize pre-existing breaks at a near physiological ionic strength (120 mM NaCl). We found that although affinity was slightly higher for the substrate with pre-existing nicks, the increase was only ∼1.6-fold (Supplementary Figure S5P–Q). Collectively, these results suggest that Mlh1-Pms1 has minimal intrinsic preference for DNA with pre-existing nicks compared with unnicked DNA and this preference is also not significantly affected by ATP, PCNA or in combination. These data also suggest that the mlh1N35-pms1N34A ATPase mutant is competent for DNA binding and is no more affected by pre-existing nicks, ATP or PCNA than the wild-type protein, which is consistent with the data in Figure 3. These data suggest that the selective protection for pre-existing nicks over endonuclease products observed in Figures 2–3 is not likely driven by affinity or ATPase activity but by an alteration in the dynamics or interactions of Mlh1-Pms1 with its own endonuclease product and a pre-existing nick.

### Mlh1-Pms1’s ATPase activity loses PCNA stimulation on DNA with pre-existing nicks

**Figure 5.**
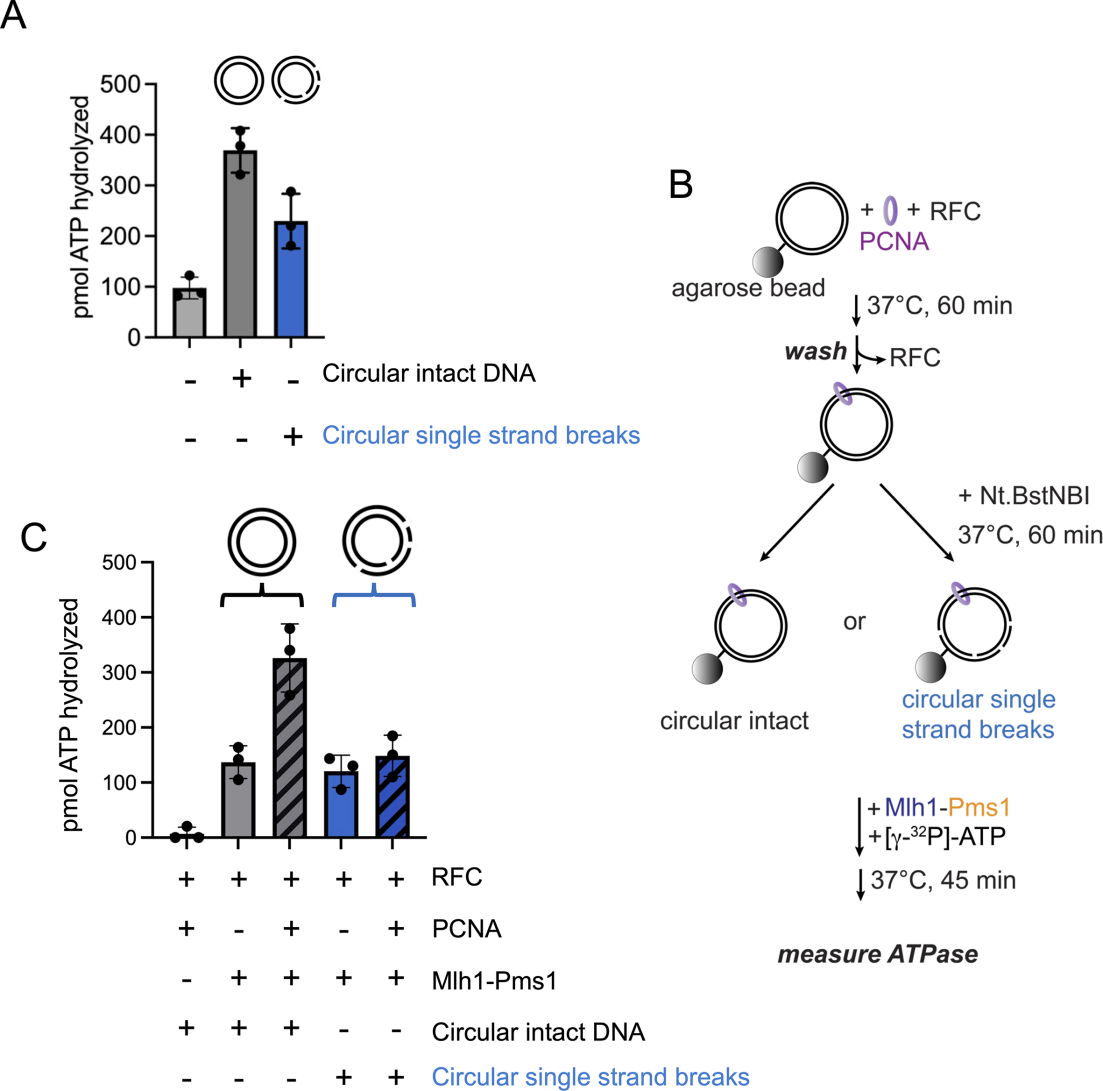
Mlh1-Pms1’s intrinsic ATPase activity loses PCNA stimulation in the presence of DNA breaks. (**A**) TLC ATPase assay performed measuring the amount of ATP hydrolyzed by Mlh1-Pms1 without DNA, on 4.3 kb relaxed, circular DNA without nicks, and circular 4.3 kb DNA containing four nicks (*n* = 3 for all conditions). 4.3 kb pBR322 was relaxed using topoisomerase I (NEB). Single-strand breaks were made using Nt.BstNBI. For the ATPase reaction (10 μl), 3.8 nM DNA, when included, was incubated with 400 nM Mlh1-Pms1 and 100 μM radiolabeled ATP in a buffer containing 20 mM KCl and 2.0 mM MgCl_2_. See ‘Materials and methods’ section for additional details. (**B**) Schematic for assay in panel (C). Biotinylated 4.3 kb plasmid was immobilized on streptavidin-linked agarose beads. PCNA was loaded by RFC and removed from the reaction. For substrates with single-strand breaks, four nicks were introduced by Nt.BstNBI (NEB). Immobilized substrates with and without (circular intact) single-strand breaks (76 fmol) were incubated with 4.0 pmol of Mlh1-Pms1 and 967 pmol of radiolabeled ATP in the buffer used in panel (A). ATPase activity was measured as in panel (A). (**C**) Amount of Mlh1-Pms1 ATPase activity on DNA substrates where PCNA was pre-loaded where indicated. Negative control in lane 1 demonstrates that RFC was efficiently removed from the reaction and did not contribute background activity.

We performed an ATPase assay to measure whether Mlh1-Pms1’s ability to hydrolyze ATP is altered in the presence of DNA with pre-existing nicks relative to DNA without nicks, taking advantage of past work demonstrating that Mlh1-Pms1’s ATPase activity is stimulated by DNA (16,28,29). We generated both relaxed 4.3 kb circular DNA and a 4.3 kb plasmid with four pre-existing nicks. We selected circular DNA so that we can control for the number of DNA breaks. This size of substrate was selected over a 2.7 kb plasmid as used in Figures 1–4, because larger DNA molecules are more stimulatory to MutL-family ATPases (50). In the presence of the unnicked 4.3 kb plasmid, ATPase activity was stimulated ∼3.8-fold compared with reactions with Mlh1-Pms1 alone. When we performed the assay with a plasmid with four pre-existing nicks, Mlh1-Pms1’s ATPase activity was stimulated by ∼2.3-fold over the negative control without DNA (Figure 5A). Because large circular DNA was used in this assay to control for nonspecific binding to DNA ends that is observed on linear DNA substrates and to ensure that we reliably measure activity, our substrate with pre-existing nicks has long continuous stretches of DNA which may contribute background stimulation. Despite these limitations, these data suggest that pre-existing nicks may inhibit Mlh1-Pms1 ATPase activity.

In addition to being stimulated by DNA, Mlh1-Pms1’s ATPase activity has also been shown to be stimulated by interactions with PCNA on DNA (16,28,29). To determine whether Mlh1-Pms1 retains this stimulation, we generated a substrate on which we could pre-load PCNA and remove RFC as a contaminating ATPase to measure Mlh1-Pms1 ATPase activities (Figure 5B). To do this, briefly, we prepared biotinylated circular DNA as described in the ‘Materials and methods’ section. We immobilized this substrate on streptavidin-conjugated agarose beads and incubated with RFC and PCNA in the presence of ATP, allowing RFC to load PCNA onto the DNA. Although not as efficient as other substrates, RFC has been shown to be able to load PCNA onto relaxed, covalently closed DNA (17). After PCNA loading, we isolated the PCNA-bound substrate through centrifugation and washed away unbound components, including RFC and the ATP present for PCNA loading. We confirmed that no residual RFC is leftover after the purification method by running a control experiment that contains RFC, PCNA and DNA (Figure 5C, column 1). After the PCNA loading step and subsequent washes, we performed the ATPase assay on the PCNA-loaded DNA in the absence of Mlh1-Pms1. We did not observe hydrolysis in this control experiment, suggesting no residual RFC is present. Previous studies also indicate that this method effectively removes all RFC, as RFC does not remain associated with DNA in the presence of PCNA and ATP (17,51). Specifically, gel filtration experiments conducted under conditions similar to ours showed that, after PCNA loading, RFC did not co-elute with plasmids containing loaded PCNA (17). After removing RFC, we divided the bead-bound DNA into portions and incubated one portion with a restriction nicking endonuclease that nicks the DNA at four specific locations. This nicking was performed after PCNA loading to ensure that RFC did not preferentially load PCNA at the nicked sites. We then incubated either the unnicked PCNA-bound DNA or the nicked PCNA-bound DNA with wild-type Mlh1-Pms1 and radiolabeled ATP under condition where we do not see endonuclease activity. Control experiments were also performed in the absence of Mlh1-Pms1 and/or the absence of PCNA.

Consistent with our data in Figure 5A, we observed that Mlh1-Pms1 was able to hydrolyze ATP in the presence of unnicked plasmid DNA (Figure 5C, lane 2). When PCNA was pre-loaded on these substrates, the amount of ATP hydrolyzed increased ∼2.4-fold relative to the amount hydrolyzed without PCNA, demonstrating that PCNA is capable of stimulating Mlh1-Pms1’s ATPase activity on these substrates as has been previously reported (Figure 5C, lane 3) (16,28). When the experiment was performed on DNA with pre-existing nicks but no PCNA, the amount of hydrolysis was ∼1.2-fold lower than when an unnicked substrate was used at this condition. When we performed the experiment with pre-nicked DNA pre-loaded with PCNA, the amount of ATP hydrolyzed did not significantly change relative to the ATPase reaction on this substrate without PCNA (Figure 5C, compare lanes 4 and 5).

It should be noted that the conditions used in this experiment are distinct from those used in Figure 5A which explains why we observe minimal inhibition in the ATPase activity by the substrate with pre-existing nicks compared with the unnicked DNA. Assays in Figure 5A and C both contain 4 pmol of Mlh1-Pms1, 967 pmol of ATP and 76 fmol of DNA, although a small amount of DNA may be lost in the assay on streptavidin-agarose beads due to the conjugation and washing steps. The reaction volume in Figure 5A is held constant at 10 μl, while the reaction volume in Figure 5C is 30 μl due to the volume of the beads in addition to the buffer and reaction components, thus the reactions in Figure 5C are more dilute than those in Figure 5A, obscuring the small inhibitory effect we observed in Figure 5A. Importantly, though, on DNAs where PCNA is pre-loaded, we lose the stimulatory effect of PCNA in the presence of pre-existing nicks. These results suggest that when pre-existing nicks are present, Mlh1-Pms1 likely has distinct interactions with that nick that do not support PCNA-stimulated or robust ATP hydrolysis.

### Mlh1-Pms1’s endonuclease activity is inhibited on DNA with pre-existing nicks

Our data suggest that Mlh1-Pms1 exhibits distinct behavior on DNA with pre-existing nicks compared with its endonuclease product after nicking and that this behavior is regulated by Mlh1-Pms1’s ATPase activity. Our data in Figures 1–2, suggest that Mlh1-Pms1 uses ATPase activity to disengage from the nick it creates, allowing it to nick DNA again. However, pre-existing nicks appear to inhibit ATPase activity, potentially preventing disengagement from these sites. To test the effect of pre-existing nicks on Mlh1-Pms1’s endonuclease activity, we performed an endonuclease assay using 4.3 kb linear plasmid-based substrates. These substrates were chosen to bypass the need for RFC, which preferentially loads PCNA onto circular DNA at nicks, while linear substrates allow PCNA to self-load onto DNA ends. Additionally, we selected a larger substrate size (4.3 kb) than those used in Figures 1–2 to ensure robust endonuclease activity, as linear DNA is a less effective substrate for Mlh1-Pms1 than equivalent-sized circular substrates. This necessitates the use of a larger DNA size for these experiments (10–12).

**Figure 6.**
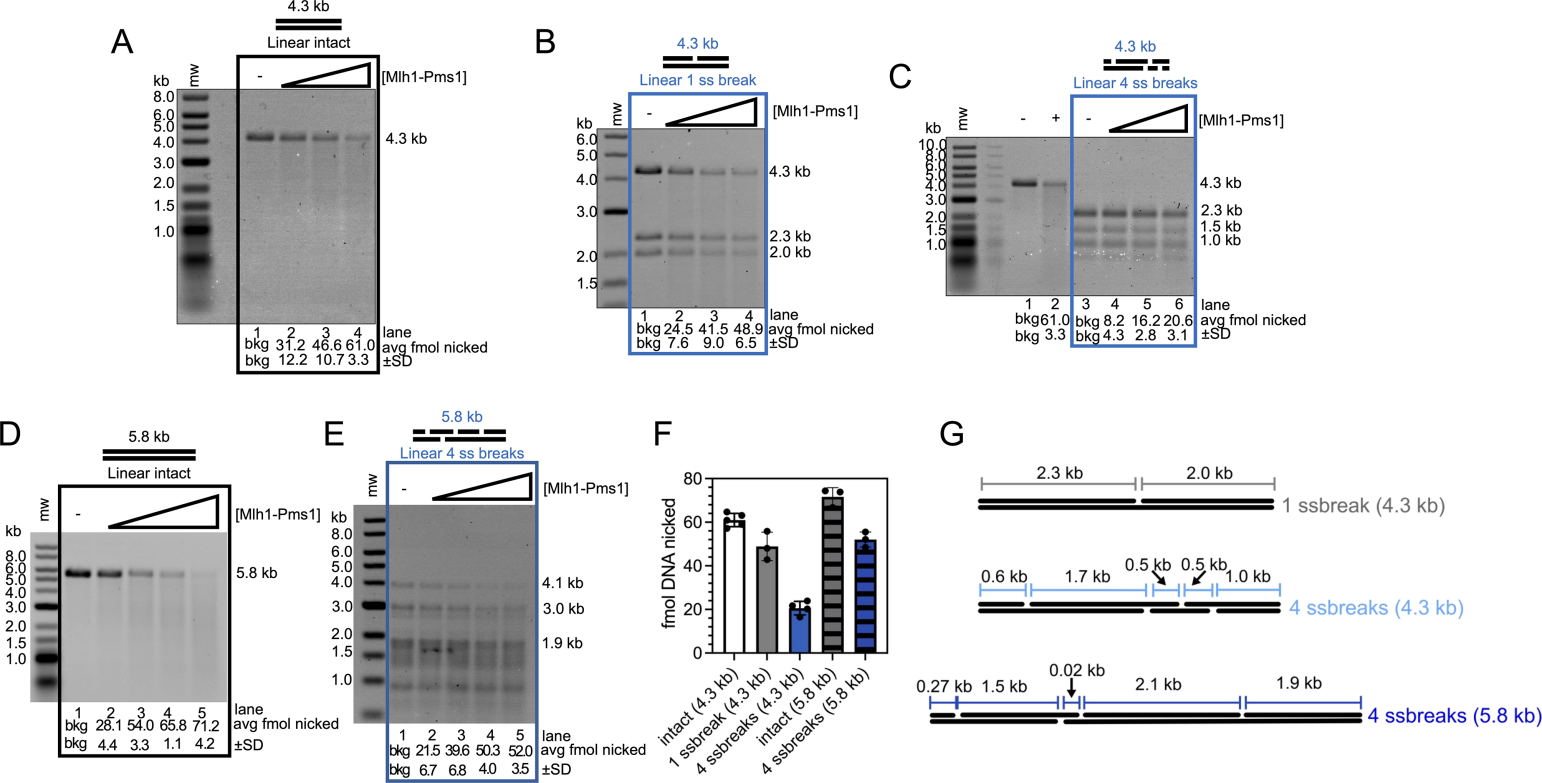
Mlh1-Pms1’s endonuclease activity is sensitive to pre-existing nicks in the absence of RFC. Endonuclease assays are performed as described in the ‘Materials and methods’ section and analyzed by denaturing gel electrophoresis. All reactions contain 0.5 mM ATP and 500 nM PCNA in a buffer containing 20 mM KCl and 2.5 mM MnSO_4_. Because endonuclease activity is nonspecific on substrates in this figure, the amount of DNA nicked is measured as the degradation of DNA bands relative to negative controls. Where indicated, 1 kb plus ladder (mw) (NEB) was used to measure starting material fragment sizes. Sizes indicated to the right of the gel are lengths of initial DNA fragments that form the substrates. (**A**) Intact linear 4.3 kb substrates incubated with increasing concentrations of Mlh1-Pms1. Lanes 1–4 contain 0, 50, 100 and 200 nM Mlh1-Pms1, respectively. The density of the band migrating at the position of the starting material was measured for each experimental lane and the amount of substrate lost was determined by comparing these densities to the density of the substrate band in the negative control lane (lane 1) and then converted to fmol relative to the fmol of DNA used for the negative control. Average amounts of substrate lost were calculated for five replicates (*n* = 5) and reported below the gel along with the S) between experiments. (**B**) Linearized 4.3 kb DNA substrates containing with one single-strand break generated with Nt.BspQI were incubated with increasing amounts of Mlh1-Pms1 as indicated in panel (A). The amount of DNA nicked was calculated as in panel (A), with the densities of each single-strand band being measured and compared with their counterpart in the negative control (lane 1). *n* = 3. (**C**) Linearized 4.3 kb substrates containing four single-strand breaks generated with Nt.BstNBI were incubated with increasing amounts of Mlh1-Pms1 as in panel (A). Lanes 1 and 2 are negative and positive controls (where +, Mlh1-Pms1 is 200 nM) with unnicked 4.3 kb substrates, respectively. Lanes 3–6 contain the same concentrations and A and B. *n* = 4. (**D**) Unnicked linearized 5.8 kb substrate was incubated with increasing amounts of Mlh1-Pms1: 25, 50, 100 and 200 nM in lanes 2–5. All other reaction conditions are identical to panels (A)–(C). *n* = 3. (**E**) Linearized 5.8 kb DNA substrate with four single-strand breaks generated by Nt.BstNBI was incubated with increasing amounts of Mlh1-Pms1 as in panel (D). *n* = 3. (**F**) The average amount of substrate molecules nicked for each substrate at the 200 nM Mlh1-Pms1 titration point. Individual trials are indicated, and the error bars represent SD between experiments. (**G**) Schematic of substrates used in this assay with distances between nicks, regardless of strand, indicated.

We monitored endonuclease activity on 4.3 kb linear substrates with and without pre-existing nicks using denaturing agarose gels. Similar to the analysis using denaturing gels in Figure 1, because nicking events are nonspecific, nicking results in an apparent depletion of the substrate. On intact linear DNA, we observed robust endonuclease activity that increased as a function of Mlh1-Pms1 concentration (Figure 6A). When we performed this experiment with the 4.3 kb linear DNA with one single-strand break, we found that Mlh1-Pms1’s endonuclease activity was slightly inhibited compared with the intact substrate (Figure 6B and F). When we increased the number of single-strand breaks on this substrate to four, we found that Mlh1-Pms1’s endonuclease was even further inhibited (Figure 6C and F). Although Mlh1-Pms1 can bind to DNA double-strand break ends (41–44), the same number of double-strand break ends was present in all reactions regardless of substrate, so this does not account for the inhibition we see as we increase the number of pre-existing nicks. Additionally, previous studies have suggested that pre-existing nicks on plasmid substrates direct the polarity of Mlh1-Pms1 endonuclease activity (14,15). In contrast, our data show apparent inhibition on pre-nicked linear DNA. These observations suggest that RFC, which is required for PCNA loading on plasmids but was not included in our linear DNA experiments, may play an additional role in directing Mlh1-Pms1 activity.

Although it should be noted that if Mlh1-Pms1 nicks near a DNA end or on substrates with pre-existing nicks near a nick, then apparent degradation will not be visible in this assay, similar nicking inhibition has previously been observed on non-continuous DNA in experiments using the meiotic homolog Mlh1-Mlh3 (11). In these experiments, Mlh1-Mlh3 displayed partially inhibited endonuclease activity on substrates with non-B-form features, such as an eight-nucleotide insertion loop. It was suggested that on these substrates, the Mlh1-Mlh3 oligomeric assembly was disrupted which led to the inhibition. Because Mlh1-Mlh3 and Mlh1-Pms1 are homologs and share a subunit, we hypothesize that pre-existing nicks may play a similar role and disrupt or terminate an Mlh1-Pms1 oligomer. As discussed previously, Mlh1-Pms1 binds to substrates ≥500 bp and requires substrates to be larger than 1 kb for efficient endonuclease activity. Although, the duplex nature of DNA maintains the molecular architecture of the model plasmid substrate, single-strand breaks may disrupt or terminate an Mlh1-Pms1 oligomer, making the substrate behave more like consecutive small DNA fragments of sizes that do not support oligomeric complexes and endonuclease activity.

To investigate this, we prepared a substrate with four pre-existing nicks but distributed differently on the substrate relative to the arrangement in Figure 6C (see Figure 6G for schematic of nick distribution). Our goal was to create a substrate where rearranging the nicks would produce a duplex region of comparable length to that of the 4.3 kb substrate with a single pre-existing nick. This required a slight increase in plasmid size, from the 4.3 kb plasmid used in Figure 6A–C to 5.8 kb.

To account for this size difference, we first measured the endonuclease activity on the linearized 5.8 kb plasmid without any pre-existing nicks and observed an increase in activity by ∼1.2-fold, consistent with past work suggesting that larger DNAs increase Mlh1-Pms1 specific activity (11,12). When we assessed the endonuclease activity on the 5.8 kb substrate with four pre-existing nicks, we found that the inhibition by these breaks was only ∼1.4-fold, compared with an inhibition of ∼3-fold on the 4.3 kb substrate where the nicks were positioned closer together.

Additionally, on the 5.8 kb substrate, the longest continuous stretch of duplex DNA was approximately 2.1 kb—longer than the longest continuous stretch on the 4.3 kb substrate (∼1.7 kb) but closer in length to the 2.3 kb continuous duplex region on the 4.3 kb substrate with a single pre-existing nick (Figure 6G). At the highest titration point, the amount of nicked product was similar between the 5.8 kb substrate with four nicks and the 4.3 kb substrate with one nick (Figure 6F). This suggests that pre-existing nicks may disrupt Mlh1-Pms1 oligomer formation, and is also consistent with prior findings that indicate linear duplexes of at least ∼2.0 kb are necessary to support measurable Mlh1-Pms1 endonuclease activity (12).

## Discussion

In this study, we demonstrate that ATP hydrolysis enables Mlh1-Pms1 to disengage from its own endonuclease sites. Although ATP had minimal effect on the affinities for circular DNAs or long linear substrates, it has been shown to promote dissociation on short oligonucleotide substrates (28). We hypothesize that this dissociation may occur through a sliding mechanism, as previously suggested, and that long, plasmid-based DNAs restrict this activity, preventing sliding and dissociation at the ends (52–54). Through generating mutant variants where ATP binding and hydrolysis is attenuated in both or individual subunits, we determined that disengagement from endonuclease products is primarily driven by hydrolysis in the Mlh1 subunit. Using exonuclease protection assays on substrates where Mlh1-Pms1 is endonuclease active and substrates with pre-existing nicks, we observed that the ATPase-deficient Mlh1-Pms1 mutant becomes stalled at its endonuclease sites, unable to release from the DNA, but that both wild-type and ATPase-deficient Mlh1-Pms1 protect DNA at pre-existing nicks from exonuclease degradation. Although Mlh1-Pms1 does not appear to display selective binding to DNA with pre-existing nicks, when present, these structures affect Mlh1-Pms1 enzymatic activities, including PCNA-stimulated ATPase activity and nonspecific endonuclease activity. Other mismatch repair factors, notably Msh2-Msh3 has also been shown to display distinct enzymatic properties in the presence of different DNA structures (55).

**Figure 7.**
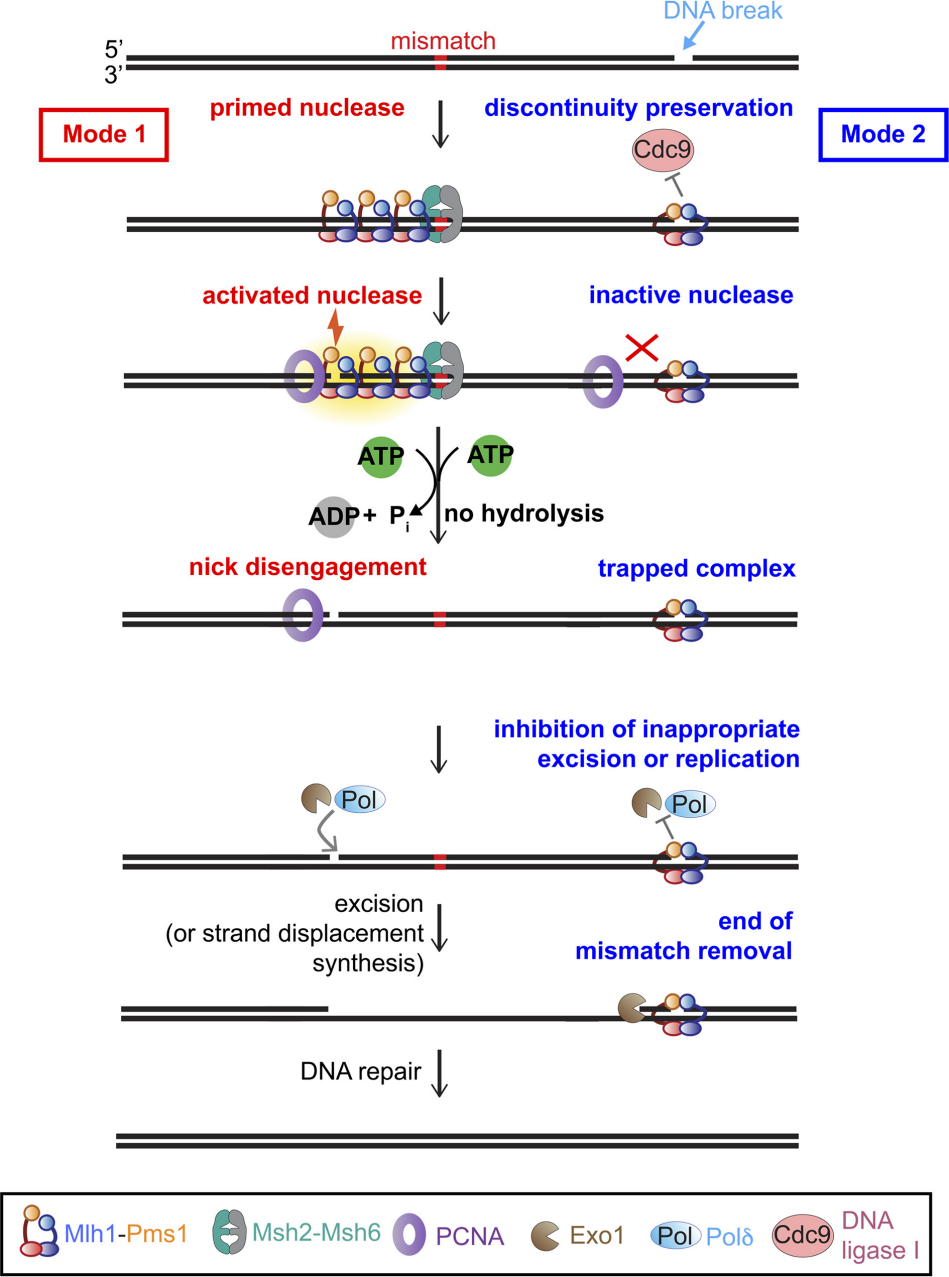
Model for Mlh1-Pms1’s distinct behaviors on nicks that are Mlh1-Pms1 endonuclease products and pre-existing nicks. In the absence of discontinuities, Mlh1-Pms1 can prime its endonuclease activity (Mode 1, left) to interact with PCNA, nick DNA, hydrolyze ATP through PCNA interactions and disengage or recycle to signal mismatch removal. In the presence of DNA breaks or discontinuities, Mlh1-Pms1 can bind to the discontinuity (Mode 2, right) and protect the break from ligation. The pre-existing nick-bound Mlh1-Pms1’s ATPase is not stimulated be PCNA, which could inhibit its endonuclease activity and attenuate disengagement from the site. This recognition can also protect breaks in the DNA that are not sites for mismatch removal, prevent to formation of more breaks introduced by Mlh1-Pms1 and may also serve as a termination point for mismatch removal. See ‘Discussion’ section.

Our results indicate that Mlh1-Pms1 exhibits distinct behaviors depending on whether it is engaging with DNA immediately after nicking or whether it encounters pre-existing nicks, suggesting two operational modes in the mismatch repair process. In post-replicative mismatch repair, Mlh1-Pms1 is recruited to an error-containing substrate that is intact in the vicinity of the mismatch. Mlh1-Pms1 is then activated to nick the nascent DNA strand through interactions with PCNA on the DNA. Interactions with PCNA, then promote the ATPase activity of the complex driven by the Mlh1 subunit, which our data suggest triggers disengagement from the Mlh1-Pms1-generated nick which could allow proteins that remove the mismatch to have access to the DNA nick (Figure 7, Mode 1, left side).

As a second mode, our data suggest that if Mlh1-Pms1 complexes encounter pre-existing DNA discontinuities, which may be present on a nascent strand of DNA as byproducts of replication and other repair processes (1,19,20), Mlh1-Pms1 may then bind to these discontinuities and is then prevented from introducing additional strand breaks and is slow or incapable of disengaging from these pre-existing nicks. Although, Mlh1-Pms1 does not display high affinity for these sites in our assay as such, as Mlh1-Pms1 searches DNA for a mispair-bound Msh2-Msh6, it may encounter these structures (56). We hypothesize that this mode could serve as a protection mechanism preventing nick ligation by Cdc9 (DNA ligase I) and preventing Polδ or Exo1 from inappropriately processing the break that is not adjacent to a mismatch, as suggested for *E. coli* MutL’s affinity for 3′-recessed ends (44). Mlh1-Pms1 bound to pre-existing nicks in this mode may also serve as a termination mechanism for mismatch removal initiating at the Mlh1-Pms1-generated nick (Figure 7, Mode 2, right side).

As mentioned earlier, in post-replicative mismatch repair, the nascent DNA strand can have discontinuities on the lagging strand between Okazaki fragments and on the leading strand from non-processive replication or other repair processes (1,19,20). Work in yeast has demonstrated that the maintenance of discontinuities on nascent DNA is necessary for efficient mismatch repair *in vivo* (21) and work in yeast, human and *Xenopus* systems demonstrates their importance *in vitro* and suggests that they serve as strand bias landmarks by acting as PCNA loading sites (4,9,14,15,17,34,57). In these models, PCNA ultimately dictates strand discrimination, but the mechanistic details of this communication channel are not established. Precisely how PCNA retains bias after being loaded at pre-existing nicks is unclear. Additionally, it is unclear *in vivo* whether the PCNA that activates Mlh1-Pms1 in mismatch repair is loaded distinctly for repair or deposited by the replication machinery since Mlh1-Pms1 does not colocalize with Msh2-Msh6 or replication proteins (18). Despite these questions for how strand discontinuities regulate mismatch repair, retaining these discontinuities as signals is important for mutation avoidance (21) and our data suggest a mechanism for how Mlh1-Pms1 protects these breaks from ligation or inappropriate processing during repair.

Our models suggest that ATP plays a critical role in driving Mlh1-Pms1 dynamics after nicking DNA and the fate of this post-nick complex is critical for downstream repair. Consistent with this, in *in vivo* assays in *S. cerevisiae* measuring mismatch repair, mutants that are defective to ATP hydrolysis have phenotypes similar to *mlh1Δ or pms1Δ* strains (27). Our data measuring the fate of an ATPase mutant on DNA after endonuclease activities suggest that these mutants remain trapped at DNA nick sites, preserving the DNA damage similar to how PARP proteins can become trapped at DNA damage sites in the presence of PARP inhibitor (58,59). This could account for the phenotype *in vivo* in bakers’ yeast. Interestingly, although both the *mlh1-N35A* and the *pms1-N34A* (reported as pms1-N65A due to numbering from a different start codon) strains gave phenotypes similar to *mlh1Δ* and *pms1Δ* haploid strains, in experiments in diploid heterozygous strains, although an *MLH1/mlh1Δ* strain has a mutation rate only slightly above wild type, an *MLH1/mlh1-N35A* strain has a rate 480-times the wild type, suggesting that the mlh1-N35A protein may be poisonous to the pathway (27). This is consistent with our data which suggest that defects in Mlh1-Pms1 ATPase activity create trapped complexes on DNA after endonuclease activity. Failures in Mlh1-Pms1 turnover are driven largely by failures in Mlh1’s ATPase activity which is required for Mlh1-Pms1 to dissociate from the nick it makes and recycle on the DNA for iterative rounds of nicking.

The ATPase motifs in Mlh1 and Pms1 are highly conserved across organisms. Based on structural work in *E. coli*, the N35 residue in the Mlh1 ATPase site and the N34 residue in the Pms1 subunit’s ATPase site are used to coordinate a divalent metal that is necessary to stabilize the negative charges on ATP (27,29). By mutating this residue and eliminating ATP binding and subsequent hydrolysis, we demonstrated a link between the ATPase activity, product recognition and recycling. This residue in particular is highly conserved among MutL homologs and missense mutations to this residue (N38 in human) among others adjacent to the ATPase site are associated with Lynch syndrome (47,48). Our data suggest that a mechanistic explanation for this disease state may be due to this protein making a nick adjacent to a mismatch but then failing to disengage from the nick that it generates. In this scenario, not only does the mismatch persist, but also additional DNA damage has been introduced by human MLH1-PMS2’s endonuclease activity, and the persistence of an MLH1-PMS2 complex may cause additional replication stress and trigger additional instability.

## Supplementary Material

gkae1253_Supplemental_File

## Data Availability

The data underlying this article are available in the article and in its online supplementary material.
