# Supplementary material for: Mlh1-Pms1 ATPase activity is regulated distinctly by self-generated nicks and strand discrimination signals in mismatch repair
Source: Nucleic Acids Res. 2024 Dec 19;53(3):gkae1253. doi: 10.1093/nar/gkae1253 (PMC11797057; doi:10.1093/nar/gkae1253)
Supplement: gkae1253_Supplemental_File [file gkae1253_supplemental_file.pdf]

SUPPLEMENTARY MATERIAL

Mlh1-Pms1 ATPase Activity is Regulated Distinctly By Self-Generated Nicks and Strand Discrimination Signals in Mismatch Repair

AUTHORS

Jonathan M. Piscitelli[1†], Scott J. Witte[1†], Yasmine S. Sakinejad[1], Carol M. Manhart[1*]

[1]Department of Chemistry, Temple University, Philadelphia, Pennsylvania, 19122, USA

* To whom correspondence should be addressed. Tel: +1 215-204-7167; Email: carol.manhart@temple.edu

[†] Joint Authors

SUPPLEMENTARY MATERIAL

SUPPLEMENTARY TABLE

Table S1

Oligonucleotide Name	Sequence (5' to 3')	Purpose
CMO175	AATGATGGAGGCGTCCATCGATGCG	Forward primer to generate N35A mutation in *MLH1*
CMO176	TCTTTGAGAGCATTTACG	Reverse primer to generate N35A mutation in *MLH1*
CMO177	ACTCGTTGATGCGAGTATAGATGCG	Forward primer to generate N34A mutation in *PMS1*
CMO178	TCTTTCACTGCAGTTGTTAAG	Reverse primer to generate N34A mutation in *PMS1*

Table S1. Oligonucleotides used in this study. Primers CMO175-178 used to generate mutations to abrogate ATPase activity in Mlh1 and Pms1 subunits**.** Q5 mutagenesis (NEB) was performed using pMH1 and pMH8 as templates (see Materials and Methods) (31).

SUPPLEMENTARY FIGURES

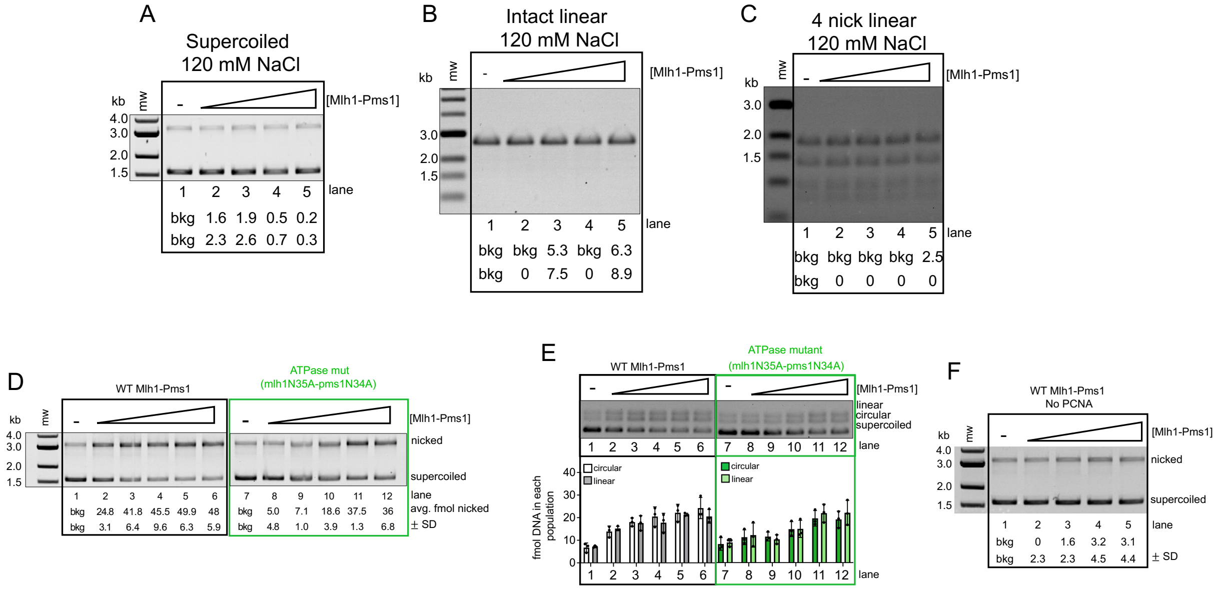

Figure S1. Wild-type Mlh1-Pms1 endonuclease activity is activated by PCNA and detectable at low ionic strength and an Mlh1-Pms1 ATPase mutant does not display delayed recycling. (A-C) Endonuclease assays using wild-type Mlh1-Pms1 with the addition of 120 mM NaCl. 0, 25, 50, 100, or 200 nM of Mlh1-Pms1 was incubated with 3.8 nM of a 2.7 kb supercoiled pUC18 DNA (A), a linear 2.7 kb plasmid without nicks (B), or a linear 2.7 kb plasmid with four pre-existing nicks (C) along with 100 nM RFC, 500 nM PCNA, and 0.5 mM ATP in a buffer containing 20 mM KCl, 120 NaCl, and 2.5 mM $MnSO_4$ to probe for endonuclease activity. See Materials and Methods for additional details. Reaction products were resolved using a native agarose gel (A) or denaturing agarose gel system (B-C). (D) Endonuclease assay using wild-type Mlh1-Pms1 or mlh1N35A-pms1N34A in the same buffer described above containing 20 mM KCl. Reactions were assembled as described in the Materials and Methods and in Figure 1A except with titrations points of 0, 25, 50, 100, 200, and 300 nM of either wild-type Mlh1-Pms1 or mlh1N35A-pms1N34A. Reactions were incubated for 2 hours at 37°C as opposed to the 1-hour incubation in Figure 1. Reaction products were resolved using native agarose conditions. (E) A portion of the reaction analyzed in panel D was resolved under denaturing conditions. (F) Endonuclease assay with wild-type Mlh1-Pms1 but excluding PCNA. 0, 25, 50, 100, or 200 nM of Mlh1-Pms1 was incubated with 3.8 nM of a 2.7 kb supercoiled pUC18 DNA along with 100 nM RFC and 0.5 mM ATP in a buffer containing 20 mM KCl and 2.5 mM $MnSO_4$ to probe for endonuclease activity. Reaction products were resolved using a native agarose gel.

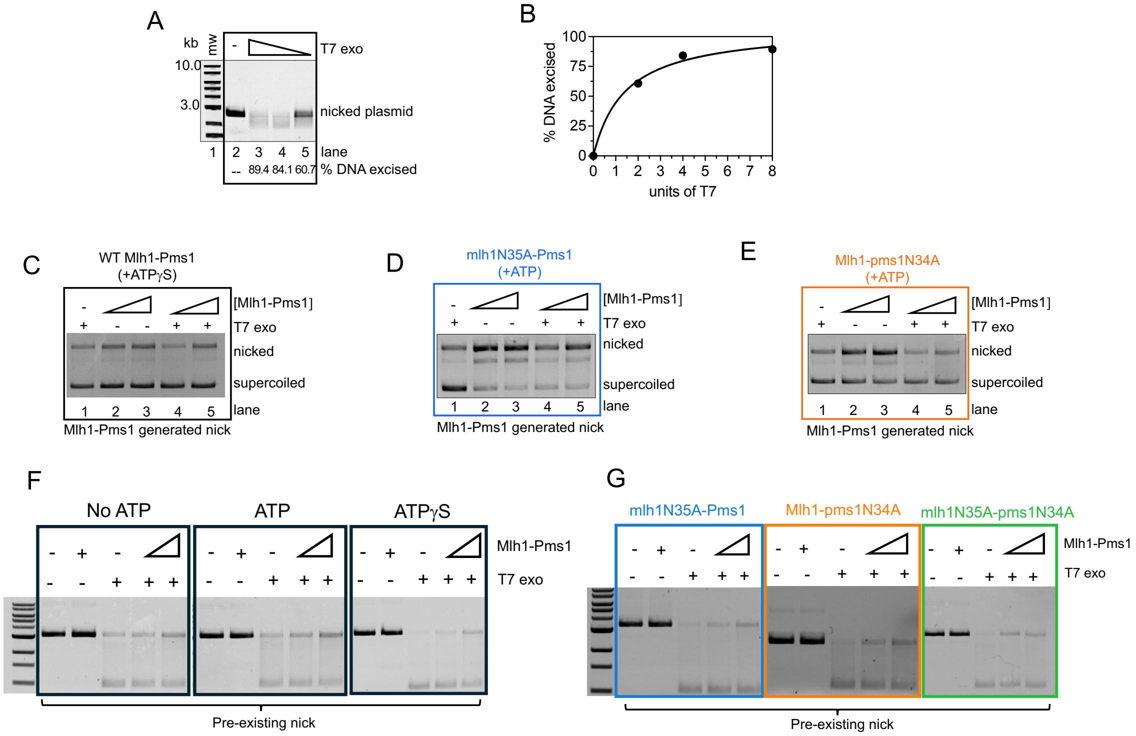

Figure S2. Optimization and sample raw data for T7 exonuclease protection assays in Figures 2-3. (A) T7 exonuclease was titrated in lanes 3-5 (2.0, 4.0, and 8.0 units) on a 2.7 kb DNA with a single pre-existing nick and analyzed by a native agarose gel to identify an amount of T7 that partially degrades nicked DNA in the absence of other factors. Top band is intact double stranded circular DNA while lower band in gel is single stranded circular DNA strand after excision. (B) Quantification of data in A fit to a hyperbolic function. The amount of DNA excised is calculated by quantifying the band density at the position of the starting material, the nicked plasmid, and determining the proportion of density that is lost compared to the negative control. This is expressed as % DNA excised. 2.0 units of T7 exonuclease was used for protection experiments in Figure 2. 4.0 units of T7 exonuclease were used in Figure 3. (C-E) Gels for T7 exonuclease protection assays where Mlh1-Pms1 generates a nick in Figure 2 using either wild-type Mlh1-Pms1 with ATPγS (C), mlh1N35A-Pms1 (D) or Mlh1-pms1N34A (E). Titration triangles indicate 50 and 100 nM Mlh1-Pms1. The amount of DNA protected in each was calculated as the proportion of nicked DNA at 50 nM and 100 nM Mlh1-Pms1 in lanes with T7 exonuclease compared to the equivalent lanes without T7 exonuclease at 50 nM and 100 nM, respectively. (F) Gels for T7 exonuclease protection assays on substrates with a pre-existing nick in Figure 3 using either wild-type Mlh1-Pms1 with no ATP, 0.5 mM ATP, or 0.5 mM ATPγS. Titration triangles indicate 50 and 100 nM Mlh1-Pms1. The lower band that appears in the lanes with the T7 exonuclease is hypothesized to be single-stranded pUC18 circular DNA. Quantification was performed by a similar method as described in panel B. (G) Gels for T7 exonuclease protection assays where Mlh1-Pms1 is incubated with plasmid with a pre-existing nick in Figure 3 using the ATPase mutants, mlh1N35A-Pms1, Mlh1-pms1N34A, or mlh1N35A-pms1N34A respectively.

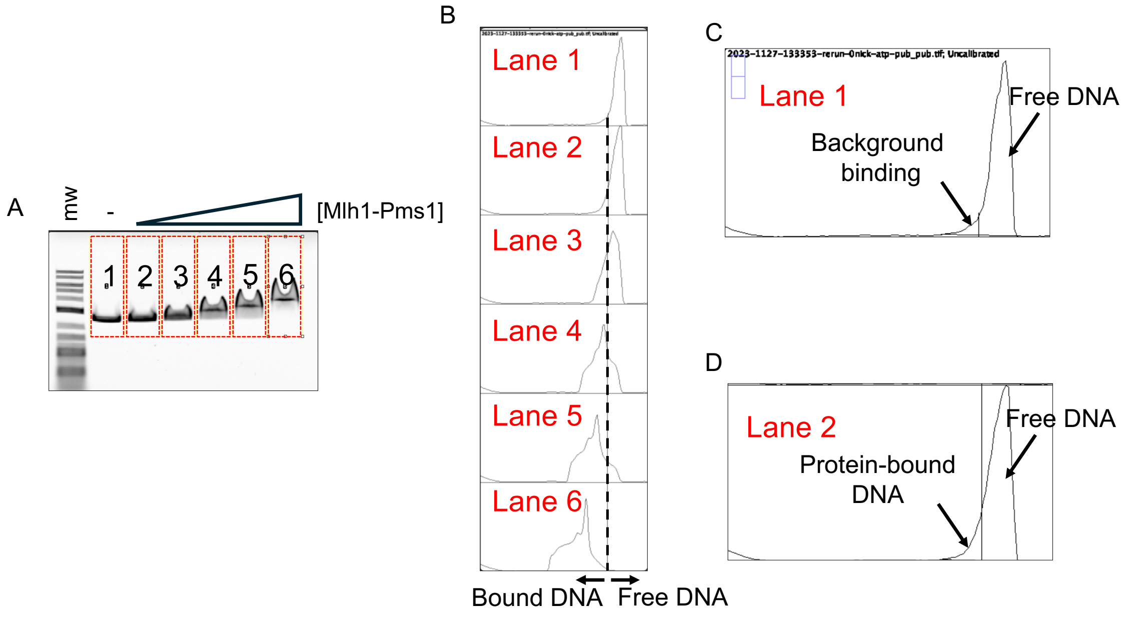

Figure S3. Quantification of bound protein from mobility shift assays. (A) Sample gel for wild-type Mlh1-Pms1 (note that this is the same gel in Figure S5B) binding to 2.7 kb linearized plasmid. Lane one contains no protein. Lanes 2-6 contain 50, 100, 200, 300, 400 nM Mlh1-Pms1, respectively. To quantify the amount of DNA bound, the entire lane is selected in ImageJ as indicated by the dotted red line. (B) Because the bound population is heterogeneous, a threshold is established using the control lane 1, where there is no protein present. The threshold is designated at the edge of the peak representing the band density in the unbound or free DNA population. This threshold is then used as cutoff to establish how much DNA is bound in each lane and how much is free by integrating the peaks to the left and right of the threshold, respectively. (C) Magnified view of the peak in lane 1 where no Mlh1-Pms1 is present. The small amount of density to the left of the threshold is quantified as "background binding" and is subtracted out of each subsequent lane. (D) Using the initial threshold as the metric for what is bound versus unbound, density to the right of the line is considered free DNA, not bound by Mlh1-Pms1. Density to the left of the designated line is considered protein-bound DNA. The percentage of DNA bound in a given lane is calculated by subtracting the amount of "background binding" in lane 1 from the amount of protein-bound DNA in that lane and dividing by the total amount of DNA present in the lane.

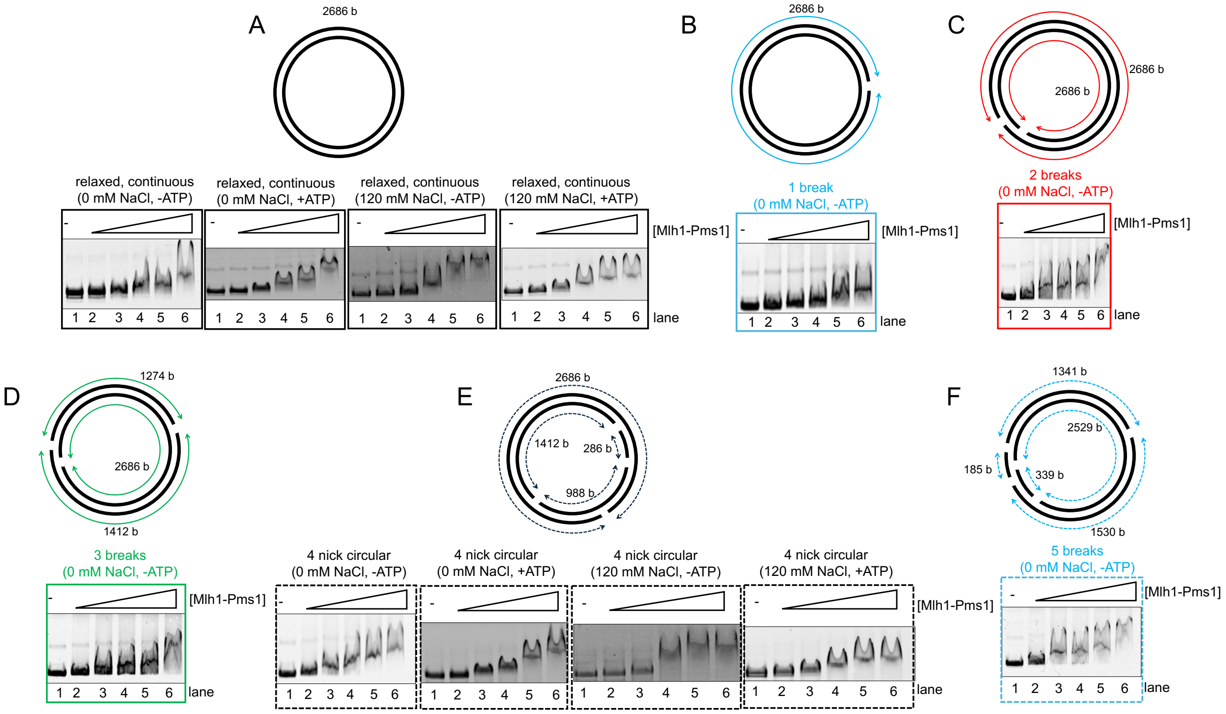

Figure S4. Mlh1-Pms1's affinity for DNA is mostly unchanged as the number of single strand breaks increases. (A-F) Representative gels for experiments in Figure 4. Mlh1-Pms1 was titrated to final concentrations: 0, 50, 100, 150, 200 or 300 nM for binding experiments on circular substrates where no NaCl was included, and no ATP was included. (A and E) representative gels for experiments in Figure 4 where the final concentrations of Mlh1-Pms1 are 0, 50, 100, 200, 300, 400 nM for binding on either a relaxed circular substrate or a circular substrate containing 4 nicks. Where indicated 0.5 mM ATP and/or 120 mM NaCl was used where indicated above the gel.

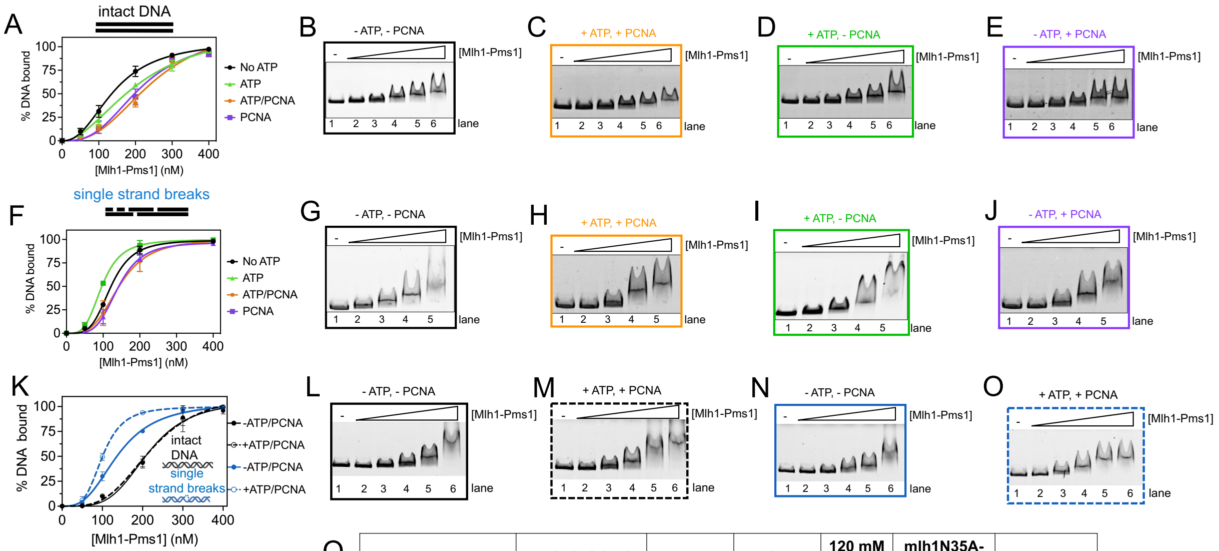

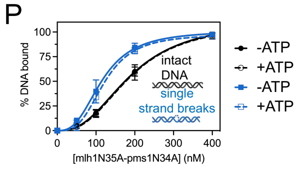

Q

DNA substrate	K_d (± SD) (nM)	ATP	PCNA	120 mM NaCl	mlh1N35A-pms1N34A	Panel
Linear Intact DNA	140 ± 17	-	-	-	-	A (B)
	210 ± 13	+	-	-	-	A (D)
	210 ± 10	-	+	-	-	A (E)
	250 ± 28	+	+	-	-	A (C)
	210 ± 29	-	-	+	-	K (L)
	220 ± 17	+	+	+	-	K (M)
	190 ± 23	-	-	-	+	P
	190 ± 42	+	-	-	+	P
Linear DNA with 4 single strand breaks	120 ± 3	-	-	-	-	A (G)
	100 ± 2	+	-	-	-	A (D)
	140 ± 4	-	+	-	-	A (J)
	140 ± 30	+	+	-	-	A (H)
	140 ± 3	-	-	+	-	K (N)
	100 ± 3	+	+	+	-	K (O)
	120 ± 15	-	-	-	+	P
	120 ± 6	+	-	-	+	P

Figure S5. Mlh1-Pms1's affinity for linearized plasmid with single strand breaks relative to intact DNA is mostly unaffected by PCNA, ATP, or an increase in ionic strength. (A) Quantification of electrophoretic mobility shift assays in panels B-E. Data were fit to a sigmoidal function modeling cooperative binding. (B-E) A 2.7 kb DNA with no nicks was linearized by HindIII and incubated with 0, 50, 100, 200, 300, or 400 nM of Mlh1-Pms1 where indicated in a buffer containing 0 mM NaCl. Where indicated 0.5 mM ATP and/or 500 nM PCNA was added. Binding was assessed as in Figure S4. (F) Quantification of electrophoretic mobility shift assays in panels G-J. Data were fit to a sigmoidal function modeling cooperative binding. (G-J) A 2.7 kb DNA was nicked four times by Nt.BstNBI, was linearized by HindIII, and incubated with 0, 50, 100, 200, or 400 nM of Mlh1-Pms1 where indicated in a buffer containing 0 mM NaCl. Where indicated 0.5 mM ATP and/or 500 nM PCNA was added. Binding was assessed as in Figure S4. (K) Quantification of electrophoretic mobility shift assays in panels L-O. Data were fit to a sigmoidal function modeling cooperative binding. (L-O) Linear 2.7 kb plasmid with or without nicks were generated as in B-E or G-J, respectively and were incubated with 0, 50, 100, 200, 300, or 400 nM of Mlh1-Pms1 where indicated in a buffer containing 120 mM NaCl. Where indicated 0.5 mM ATP and/or 500 nM PCNA was added. (P) Quantification of electrophoretic mobility shift assays with mlh1N35A-pms1N34A on intact linear 2.7 kb DNA and linear DNA with single strand breaks generated as described above in a binding reaction at 0 mM

NaCl. Where indicated, 0.5 mM ATP was added. The mlh1N35A-pms1N34A protein was titrated to final concentrations of 0, 50, 100, 200, or 400 nM. (Q) Table of all estimated K_d values from figures (A, F, K, and P). n = 3 for all conditions.
